# DNAJB4 Protects Against Atherosclerosis by Stabilizing the HSP70-LXRα Axis in Hepatic Lipid Metabolism

**DOI:** 10.7150/ijbs.131807

**Published:** 2026-08-21

**Authors:** Wen-Hua Chen, Chia-Hui Chen, Man-Chen Hsu, Po-An Hu, Hsueh-Te Lee, Hsuan-Yun Hu, Kang-Yi Su, Wei-Lun Hsu, Tzong-Shyuan Lee

**Affiliations:** 1 Graduate Institute and Department of Physiology, College of Medicine, National Taiwan University, Taipei 10051, Taiwan; 2 Institute of Anatomy & Cell Biology, College of Medicine, National Yang Ming Chiao Tung University, Taipei 112304, Taiwan; 3 Taiwan International Graduate Program in Molecular Medicine, National Yang Ming Chiao Tung University and Academia Sinica, Taipei 112304, Taiwan; 4 Department of Clinical Laboratory Sciences and Medical Biotechnology, College of Medicine, National Taiwan University, Taipei 10051, Taiwan; 5 Department of Laboratory Medicine, National Taiwan University Hospital, Taipei 100225, Taiwan

**Keywords:** DNAJB4, HSP40, HSP70, LXRα, atherosclerosis

## Abstract

DNAJB4, a member of the DNAJ/HSP40 family, functions as a co-chaperone of HSP70, regulating protein homeostasis and cellular functions. However, the molecular mechanism underlying the biological effect of DNAJB4 on lipid metabolism remains unclear. We investigated the role of DNAJB4 and its molecular mechanism in hyperlipidemia and atheroprone apolipoprotein E-null (*apoe^-/-^*) mice. Western blot analysis and immunohistochemistry were used to assess decreased DNAJB4 expression in *apoe^-/-^* mice. Moreover, the genetic deletion of DNAJB4 led to an increase in hepatic lipid accumulation and hyperlipidemia in *apoe^-/-^* mice, as evidenced by decreased expression of proteins related to cholesterol esterification and clearance, and an increased hepatic level of triglycerides, fatty acids, glycerol, free cholesterol, total cholesterol, and bile acid. Mechanistically, DNAJB4 deficiency impaired the protein stability of HSP70, reduced nuclear HSP70 association, and downregulated HSP70-induced LXRα transcription. The genetic deletion of DNAJB4 also promoted LXRα protein degradation and reduced LXRα autoregulation, thereby exacerbating the decrease in LXRα and LXRα-mediated gene expression. Furthermore, treatment with curcumin and andrographolide, the inducers of DNAJB4, did not reduce the atherosclerotic lesions at the aortic sinus in *apoe^-/-^dnajb4^-/-^* mice, suggesting that DNAJB4 is required for the atheroprotective effect of curcumin and andrographolide. Our findings indicate that DNAJB4 plays a crucial role in regulating the HSP70-LXRα axis in hyperlipidemia, hepatic lipid accumulation, and atherosclerosis. Here, we identify DNAJB4 as a critical HSP70 co-chaperone that stabilizes HSP70, promotes its nuclear association, and sustains LXRα autoregulation in hepatic lipid metabolism.

## Introduction

DnaJ heat shock protein family (HSP40) member B4 (DNAJB4) contains a J-domain protein and functions as a co-chaperone of HSP70 1. As part of the molecular chaperone system, DNAJB4 facilitates substrate transfer to HSP70 and enhances protein refolding by stimulating HSP70 ATPase activity 2, 3. Moreover, Johnson et al. reported that DNAJB4 competes with E3 ubiquitin ligases for binding to HSP70, thereby limiting ubiquitination of HSP70-bound client proteins 4, suggesting that DNAJB4 not only aids protein folding but also helps stabilize specific client proteins by preventing premature degradation. In addition to its role as an HSP70 co-chaperone, DNAJB4 acts as a tumor suppressor, inhibiting the proliferation, migration, and invasion of cancer cells by deregulating key signaling pathways involved in cell cycle regulation and epithelial-mesenchymal transition in breast, lung, and colorectal cancers 5-8. Despite its well-characterized roles in proteostasis and tumor suppression, the biological impact of DNAJB4 in lipid metabolism and lipid-associated disorders remains poorly understood. Further investigation is required to clarify the role of DNAJB4 in the pathogenesis of hyperlipidemia-induced atherosclerosis.

Hyperlipidemia, primarily caused by dysregulated hepatic lipid metabolism, is a major risk factor for the initiation and progression of atherosclerosis 9, 10. The liver is the central organ responsible for maintaining lipid homeostasis in humans 11. Circulating cholesterol levels are tightly regulated by hepatic lipid metabolic pathways, including lipoprotein internalization, *de novo* lipogenesis, cholesterol esterification, and the secretion of very-low-density lipoprotein (VLDL) and high-density lipoprotein (HDL) 12. Notably, liver X receptor α (LXRα), a transcription factor, plays a critical role in hepatic lipid metabolism and the progression of atherosclerosis 13-15. Mechanistically, LXRα functions as an auto-regulatory transcription factor that binds to LXR response elements, which contain variations of the repeated sequence AGGTCA, to activate the expression of downstream genes involved in cholesterol metabolism, such as ATP-binding cassette transporter sub-family A member 1 (ABCA1), ABCG1, ABCG8, ABCG5, and cholesterol 7α-hydroxylase (CYP7A1) 16, 17. Impaired LXRα function can lead to elevated circulating LDL levels or reduced HDL-mediated cholesterol transport, disrupting hepatic lipid metabolism 16. Recently, Gungor et al. demonstrated that HSP70 interacts with the LXRα promoter in macrophages, thereby enhancing LXRα expression and that of its target genes, thereby promoting cholesterol efflux 18. While HSP70 is known to interact with the LXRα promoter in macrophages, the specific upstream co-chaperone governing this mechanism in the liver has remained elusive. Importantly, the interplay among DNAJB4, HSP70, and LXRα in hepatic lipid metabolism and hyperlipidemia-induced atherosclerosis has yet to be fully elucidated.

Given the interplay between protein quality control and metabolic regulation, further investigation is necessary to clarify the precise role of DNAJB4 in lipid metabolism and the development of hyperlipidemia-induced atherosclerosis. To address the role of DNAJB4 in lipid metabolism, wild-type (WT) C57BL/6 mice, *apoe^-/-^* mice, *dnajb4^-/-^* mice, and *apoe^-/-^dnajb4^-/-^* mice were used to investigate the molecular mechanism of DNAJB4 in hepatic lipid metabolism and hyperlipidemia-induced atherosclerosis. Our findings demonstrate that genetic deletion of DNAJB4 exacerbates hepatic lipid accumulation and accelerates atherosclerosis progression in *apoe^-/-^* mice. Mechanistically, the genetic deletion of DNAJB4 promotes the degradation of HSP70 and LXRα, thereby reducing nuclear HSP70 association and downregulating HSP70-induced LXRα transcription. This leads to decreased autoregulation of LXRα, ultimately resulting in decreased LXRα-mediated expression of proteins involved in cholesterol metabolism. Moreover, the atherosclerotic lesion was not improved in *apoe^-/-^dnajb4^-/-^* mice treated with curcumin and andrographolide, the inducer of DNAJB4 19, 20, suggesting the crucial role of DNAJB4 in the atheroprotective effect of curcumin and andrographolide. The genetic deletion of DNAJB4 impairs the HSP70-LXRα axis and LXRα-mediated cholesterol metabolism, thereby deregulating hepatic cholesterol metabolism and promoting hyperlipidemia, ultimately accelerating atherosclerosis progression.

## Materials and methods

### Reagents

Cycloheximide (CHX), curcumin, andrographolide, and MG-132 were purchased from Cayman Chemical (Ann Arbor, MI, USA). Oil red O (O0625-25G) was acquired from Merck (Darmstadt, Germany). Rabbit antibodies for albumin, acetyl-Coenzyme A acetyltransferase 2 (ACAT2, 14755-1-AP), DNAJB4 (13064-1-AP), and mouse antibodies for β-actin were obtained from Proteintech (Rosemont, IL, USA). Rabbit antibodies for ABCG1 (ab52617), LDL receptor-related protein 1 (LRP1, ab92544), low-density lipoprotein receptor (LDLR, ab52818), and ABCG8 (ab223056); rat antibody for F4/80 (ab6640); mouse antibodies for ABCA1 (ab18180), HSP70 (ab2787), and LXRα ab41902); 4′,6-diamidino-2-phenylindole (DAPI); high-sensitivity chromatin immunoprecipitation (ChIP) kits (ab185913) were obtained from Abcam (Cambridge, MA, USA). Rabbit anti-lysosomal acid lipase (LAL; GTX101169) antibody was obtained from GeneTex (Irvine, CA, USA). Rabbit antibodies for scavenger receptor (SR)-BI (sc-67098), CYP7A1 (sc-518007), and ABCG5 (sc-25796); mouse antibody for von Willebrand factor (vWF, sc-365712) were from Santa Cruz Biotechnology (Santa Cruz, CA, USA). Rabbit antibody against neutral cholesterol ester hydrolase 1 (NCEH1, #PA5-50285) was obtained from Thermo Fisher Scientific Inc. (Waltham, MA, USA). Dil-labeled oxidized LDL was purchased from Biomedical Technologies (Stoughton, MA, USA). Kits for proximity ligation assay (PLA) and Duolink *in situ* detection reagents green (DUO92014-30RXN) were obtained from Sigma-Aldrich (St Louis, MO, USA). Cholesterol, high-density lipoprotein-cholesterol (HDL-c), and triglyceride assay kits were purchased from Randox (Crumlin, Antrim, UK). Horse anti-rabbit IgG antibody (H + L), DyLight® 488 (DI-1088) was obtained from Vector Laboratories (Burlingame, CA, USA). ELISA kits for Tumor necrosis factor α (TNF-α, MTA00), interleukin 6 (IL-6, M6000B-1), monocyte chemotactic protein 1 (MCP-1, MJE00), macrophage inflammatory protein 2 (MIP-2, MM200), and IL-1β (MLB00C-1) were from R&D Systems (Minneapolis, MN, USA).

### Animal

This study corresponds to the Guide for the Care and Use of Laboratory Animals (Institute of Laboratory Animal Resources, eighth edition, 2011), and all animal experiments were approved by the Institutional Animal Care and Use Committee (IACUC) of the College of Medicine, National Taiwan University (No. 20210257). Male *apoe*^-/-^ mice were purchased from Jackson Laboratory (Bar Harbor, Maine). Male wild-type (WT) C57BL/6 and *dnajb4^-/-^* mice were obtained from the Laboratory Animal Center of the National Taiwan University College of Medicine (Taipei, Taiwan). The *apoe*^-/-^*dnajb4*^-/-^ mice were generated by cross-breeding *apoe*^-/-^ and *dnajb4*^-/-^ mice, and the polymerase chain reaction (PCR) of genomic DNA was performed to confirm *apoe*^-/-^ and *dnajb4*^-/-^ genotypes. PCR was performed with the primers: DNAJB4 (forward): 5'-GGC TTG CTG TCT AAG GTG ATG-3', DNAJB4 (reverse): 5'-TGC CGG TTG AAA ACC AAG TC-3', DNAJB4 (mutant reverse): 5'-ACG TTC AAA GCT ATC ATA GTC-3', at 95 °C for 5 min, followed by 40 cycles at 95 °C for 30 sec, 62 °C for 30 sec, 72 °C 30 sec and additional extension at 72°C for 5 min; apoE (forward): 5'-GCC TAG CCG AGG GAG AGC CG-3', apoE (reverse): 5'-TGT GAC TTG GGA GCT CTG CAG C-3', apoE (mutant reverse): 5'-GCC GCC CCG ACT GCA TCT-3' at 95 °C for 2 min, followed by 30 cycles at 94 °C for 30 sec, 57 °C for 30 sec, 72°C 90 sec and additional extension at 72 °C for 10 min. The *apoe^-/-^* mice and *apoe^-/-^dnajb4^-/-^* mice were sacrificed by CO_2_ at five months of age. At the end of the experiments, hearts and livers were collected and subjected to further experiments.

### Cell culture

Huh7 cells were cultured with Dulbecco's modified Eagle's medium (DMEM) (Thermo Fisher Scientific, Waltham, MA) with a high glucose concentration (4500 mg/L) and supplemented with 10% FBS (Thermo Fisher Scientific) and penicillin (100 U/ml)/streptomycin (100 µg/ml) (HyClone, Logan, UT, USA). Human microvascular endothelial cells (HMECs) were obtained from the Centers for Disease Control (Atlanta, GA, USA). HMECs were maintained in DMEM supplemented with 5 % fetal bovine serum (FBS), 100 U/mL penicillin, and 100 µg/mL streptomycin. Additionally, the culture medium included 20 % Endothelial Cell Growth Medium MV2 (Promocell, Heidelberg, Germany), which contained 5 % fetal calf serum, 5 ng/mL epidermal growth factor (EGF), 10 ng/mL basic fibroblast growth factor (bFGF), 20 ng/mL insulin-like growth factor (IGF), 0.5 ng/mL vascular endothelial growth factor 165 (VEGF165), 1 µg/mL ascorbic acid, and 0.2 µg/mL hydrocortisone. The human monocytic cell line THP-1 (Bioresource Collection and Research Center, Hsinchu, Taiwan) was cultured in RPMI 1640 medium supplemented with 10% fetal bovine serum (FBS), 100 U/mL penicillin, and 100 μg/mL streptomycin (HyClone, Logan, UT, USA). THP-1 cells were induced with 50 nM PMA for five days to differentiate into macrophages. Cells were cultured in a humidified cell culture incubator at 37 °C, 95% air, and 5% CO_2_. The growth media were replaced every other day.

### Liquid chromatography-tandem mass spectrometry (LC-MS/MS) analysis

Mouse liver samples were lysed in immunoprecipitation lysis buffer, and proteomic analysis was performed by a commercial service provider (Visual Protein Company, Taipei, Taiwan). Equal amounts of protein samples were digested using trypsin and analyzed by LC-MS/MS. Peptide mass spectra and fragmentation spectra were acquired using a Thermo Scientific™ Orbitrap Fusion™ Lumos™ Tribrid™ Mass Spectrometer (Thermo Fisher Scientific, UK). For protein identification and label-free peptide quantification, MS/MS spectra were processed using Proteome Discoverer (version 2.2; Thermo Fisher Scientific, UK) with Mascot and searched against the UniProt *Mus musculus* database. Protein identifications were filtered at a false discovery rate (FDR) of 0.05%. Protein quantifications were calculated by unique peptide intensity and normalized to the total peptide amount. Differentially expressed proteins were subjected to functional enrichment analysis using FunRich (Version 3.1.3) and Ingenuity Pathway Analysis (IPA, version 76765844M) to generate pathway maps and molecular connections between control and experiment groups. Significant (p<0.05) protein relationship tables were created based on associated processes. Canonical pathway enrichment in IPA was evaluated based on -log(p-value), and pathways with -log(p-value) > 1.3 were considered statistically significant.

### Protein-protein interaction (PPI) analysis

The PPI network of the identified targets was constructed using the STRING database (version 12.0, https://string-db.org/) **21**. The species was restricted to “*Mus musculus”* with a minimum required interaction score of 0.4 (medium confidence) to ensure the reliability of the functional associations. To enhance network clarity, disconnected nodes were excluded from the final visualization.

### Primary hepatocyte isolation

Primary hepatocytes were isolated from 8- to 12-week-old male mice by a two-step collagenase perfusion method. The portal vein was cannulated with a 24G needle, and the liver was perfused with a Ca^2+^-and Mg^2+^-free buffer (HBSS pH 7.4, plus 0.5 mM EGTA) at an 8 ml/min rate for 10 min at 37°C. Next, a collagenase solution (HBSS with 5 mM CaCl2, supplemented with 0.025% collagenase type IV) was perfused at 8 ml/min for 30 min at 37°C to digest the liver. After digestion, the liver was placed in a Petri dish with 10 ml of cold hepatocyte medium. Hepatocytes were released from the liver capsule by tearing the liver lobes with two pairs of forceps and gently shaking the tissue under a tissue culture hood to obtain a cloudy cell suspension. This suspension was then filtered through a 100 µm cell strainer into a 50 ml tube. Centrifuge the hepatocyte suspension at 500 rpm for 2 min at 4°C to pellet the cells. After centrifugation, gently resuspend the cell pellet with 25 ml of William's E medium containing 10% FBS, penicillin (100 U/ml)/streptomycin (100 μg/ml), and layer over 20 ml of isolated Percoll. Mix the Percoll-cell suspension by inversion and centrifuge at 650 rpm for 5 min at 4 °C. Intact hepatocytes were washed with William's E medium by centrifugation at 500 rpm for 2 min at 4 °C. Cells were resuspended in William's E medium, and cell count was performed by trypan blue exclusion assay. Cells were seeded at 6.25 x 10^5^ cells in a 35 mm dish in William's E medium. The primary hepatocytes were cultured at 37 °C in a humidified 5% CO_2_ incubator with daily medium renewal and subjected to the experiment within 24 hr.

### Quantitative real-time PCR (qRT-PCR)

Total RNA was isolated from cells using NucleoZOL (Takara Bio USA, Inc., CA, USA) and converted into cDNA with the ToolsQuant II Fast RT kit (BIOTOOLS Co., Taipei, Taiwan). cDNAs were then used as templates for qRT-PCR. qRT-PCR was performed using a SYBR probe-based real-time quantification system (BIOTOOLS Co., Taipei, Taiwan). The targeted mRNA level was normalized to the housekeeping gene glyceraldehyde-3-phosphate dehydrogenase (GAPDH). qRT-PCR was performed with the primers: mouse GAPDH (forward): 5′- CAT CAC TGC CAC CCA GAA GAC TG-3′ and mouse GAPDH (reverse): 5′- ATG CCA GTG AGC TTC CCG TTC AG-3′; mouse LXRα (forward): 5′-AAA CAG CTC CCT GGC TTC CT-3′ and mouse LXRα (reverse): 5′-GTA CCT CCG TGA CGT CTC CA-3′.

### Histological examination

The livers harvested from mice were fixed in 10% neutral-buffered formalin, then immersed and embedded in paraffin. The tissue blocks were sectioned at 8 μm. For histological examination, heart and liver sections were deparaffinized in Hemo-De and rehydrated sequentially through graded alcohols (100%, 95%, 70%, 50%, and 30%). Deparaffinized sections were subjected to hematoxylin and eosin (H&E) staining. In quantifying atherosclerotic lesions, 50 serial sections from the aortic sinus of each mouse were collected. Ten to twelve sections, sampled from every three consecutive sections, were deparaffinized and subjected to H&E staining. To assess hepatic lipid accumulation, livers were sectioned at cryostat temperature. The livers were freshly embedded in optimal cutting temperature compound and sectioned at 10 μm. The frozen sections were washed with PBS and fixed in formalin-calcium solution (1% calcium chloride in 10% neutral-buffered formalin, pH 4) for 60 min. The sections were rinsed in ddH_2_O and 50% isopropanol for 5 min and stained with Oil Red O for 30 min. The photomicrographs of heart and liver sections were taken under the Motic TYPE 102M microscope (Motic Images Plus 2.0, Xiamen, China).

### Immunohistochemistry

The deparaffinized sections were incubated with retrieval buffer for 10 min at 37 °C. The sections were blocked with 2% BSA for 60 min at 37 °C, incubated with the primary antibody for 2 h at room temperature, and then incubated overnight at 4 °C with the corresponding horseradish peroxidase (HRP)-conjugated secondary antibody. The images were acquired using a Zeiss LSM880 confocal microscope with Zen software. The nuclear and cytoplasmic fluorescence intensities were quantified using ImageJ, and the nuclear regions were defined by DAPI staining.

### Western blot analysis

Huh7 cells, plasma, or the livers harvested from mice were lysed in immunoprecipitation lysis buffer (50 mM Tris pH7.5, 5 mM EDTA, 300 mM NaCl, 1% Triton X-100, 1 mM phenylmethylsulfonyl fluoride, 10 µg/mL leupeptin, 10 µg/mL aprotinin, and phosphatase inhibitor cocktail II and III). The total protein was extracted by centrifuging at 10000 rpm for 5 min at 4 °C. To prepare cytoplasmic and nuclear extracts, cells were lysed in hypotonic lysis buffer (10 mM HEPES, pH 7.9, 10 mM KCl, 1.5 mM MgCl2, 0.5% NP-40, 1 mM phenylmethylsulfonyl fluoride, 10 µg/mL leupeptin, 10 µg/mL aprotinin) on ice for 5 min. Cytoplasmic extracts were collected as the supernatant after centrifugation at 10000 rpm for 10 min at 4 °C. Nuclei were then lysed in immunoprecipitation lysis buffer kept on ice and drawn through a 30G insulin syringe 10 times. After an additional 15 min incubation on ice, the sample was centrifuged at 13000 rpm for 10 min at 4 °C. The supernatant was collected as the nuclear fraction. The proteins in the lysates were separated by electrophoresis on an 8%-12% SDS-polyacrylamide gel and then transferred to a PVDF membrane (Millipore, Bedford, MA). The membrane was blocked with 5% skim milk for 1 h at room temperature. The blots were then incubated with the primary antibodies and the corresponding HRP-conjugated secondary antibodies. The protein bands were visualized with an enzyme-linked chemiluminescence detection kit (PerkinElmer, Waltham, MA, USA), and band density was quantified using TotalLab 1D (TotalLab, Newcastle upon Tyne, UK).

### Hepatic lipid analysis

The lipids in the liver (about 0.01 g) were extracted by n-hexane/isopropanol (3:2, v/v) for 2 h on a shaker. The levels of triglycerides, total cholesterol, HDL-c, and non-HDL-c were measured using fluorescence assay kits (BioVision, Milpitas, CA, USA) according to the manufacturer's instructions. The fluorescence intensity was measured at an excitation wavelength of 530 nm and an emission wavelength of 590 nm using a SpectraMax i3x fluorometer (Molecular Devices, San Jose, CA, USA).

### Proximity ligation assay (PLA)

Liver sections were incubated with Duolink blocking buffer at 37℃ for 1 h, and experiments were assessed according to the manufacturer's instructions. Samples were incubated with diluted primary antibodies of rabbit anti-DNAJB4 and mouse anti-LXRα, and then incubated with specific PLA probes against rabbit or mouse IgG at 37 °C for 2 h. A ligation mix was incubated with the samples at 37 °C for 30 min to form a closed circle between two hybridized oligonucleotides. Samples were then incubated with an amplification mix at 37 °C for 2 h. The coverslips were mounted on microscope slides with DAPI mounting medium. Images of the PLA assay were acquired using a Zeiss LSM880 confocal microscope with Zen software.

### Small-interfering RNA (siRNA) transfection

Huh7 cells were seeded in 3.5 cm dishes and starved for 6 h in serum-free DMEM. After starvation, cells were transfected with either scramble or DNAJB4 siRNA using Lipofectamine RNAiMAX reagent for 24 h. The medium was replaced with fresh and regular DMEM, and the cells were subjected to chromatin immunoprecipitation (ChIP) analysis.

### Co-immunoprecipitation

Huh7 cells were lysed with NP-40 lysis buffer (10 mM HEPES, 10 mM KCl, 1,5 mM MgCl_2_, 0.5% NP-40, 0.02% sodium azide in PBS) containing protease inhibitors (1 mM phenylmethylsulfonyl fluoride, 10 µg/mL leupeptin, 10 µg/mL aprotinin) and phosphatase inhibitor cocktail II and III. After centrifuging at 10000 rpm for 5 min at 4 °C, a 1000 μg supernatant was incubated overnight with LXRα antibody and then for 4 h with protein A/G-PLUS-Agarose at 4 °C. The immune complexes were precipitated by centrifugation at 1200 rpm for 3 min and washed three times with ice-cold PBS. Pellets were resuspended and boiled in SDS lysis buffer (1% Triton, 0.1% SDS, 0.2% sodium azide, 0.5% sodium deoxycholate, 1 mM PMSF, 10 µg/mL aprotinin, and 10 µg/mL leupeptin) for 5 min. Samples were heated at 95 °C for 10 min and examined by western blot analysis.

### Chromatin immunoprecipitation (ChIP)-PCR

Livers or Huh7 cells were fixed and lysed, and were assessed according to the manufacturer's instructions. Equal amounts of cross-linked chromatin from each group were utilized for immunoprecipitation. The lysate was sonicated and incubated overnight at 4 °C with an anti-HSP70 antibody or a normal IgG negative control antibody. The immunoprecipitated DNA was subsequently analyzed by PCR; the amplified DNA products were examined by agarose gel electrophoresis. The specific band intensities were quantified using ImageJ software. *LXRα* gene promoter binding with HSP70 was performed with the primers: mouse LXRα (forward): 5′-CCA CGT GCT TTC TGC TGA GT-3′; mouse LXRα (reverse): 5′-TGC CGC GAC TAG TTC CTT TT-3′; human LXRα (forward: 5′-CTA GTG GGG AGA GCT TCT TGG-3′; human LXRα (reverse): 5′-CTC CTT ACC CAG CGC TCT TAG-3′.

### Statistical analysis

Results were presented as the mean ± standard error of the mean (SEM). For comparisons between two independent groups, statistical significance was determined using Mann-Whitney U test. To evaluate the efficacy of individual drugs across different genotypes without comparing the drugs against each other, a two-way ANOVA with a genotype-by-treatment interaction term was performed, followed by Dunnett's *post-hoc* multiple comparisons test, which compares each drug treatment specifically to the vehicle control. All statistical analyses were performed using SigmaPlot version 12.0 (Systat Software, San Jose, CA, USA). A P value of < 0.05 was considered statistically significant. Pathway significance was determined using Fisher's exact test in IPA, and pathways with -log(p-value) > 1.3 were considered statistically significant.

## Results

### Expression of DNAJB4 in the liver of WT and apoe^-/-^ mice

**Figure 1:**
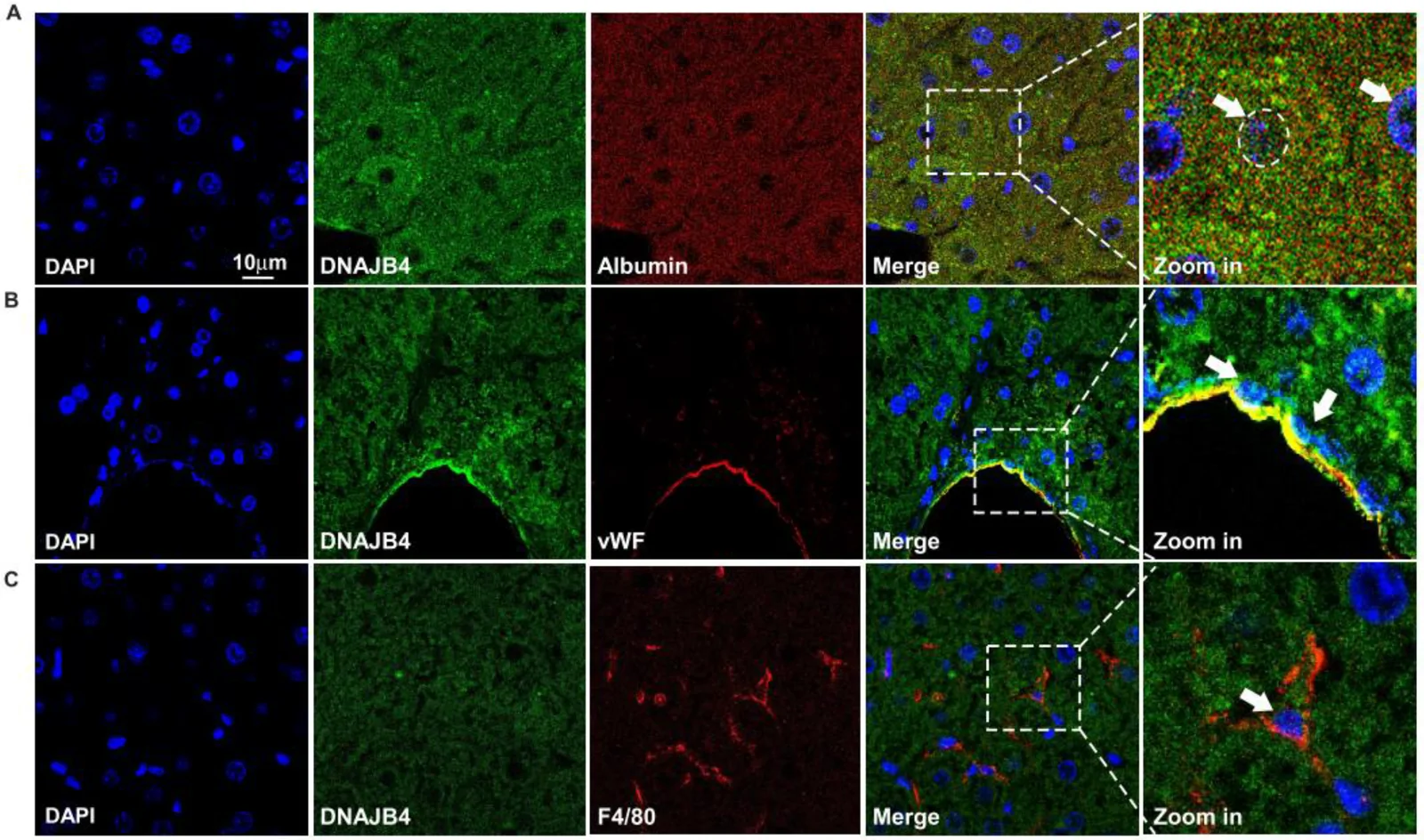
** Distribution of DNAJB4 in the liver of wild-type (WT) mice.** Five-month-old male wild-type (WT) C57BL/6 mice fed with a regular chow diet were sacrificed. Immunohistochemistry of DNAJB4 (green) and (A) albumin (red), (B) vWF (red), and (C) F4/80 (red) in the liver of WT mice. The nucleus was stained with DAPI. The arrows indicated the expression of DNAJB4 in the nucleus.

**Figure 2:**
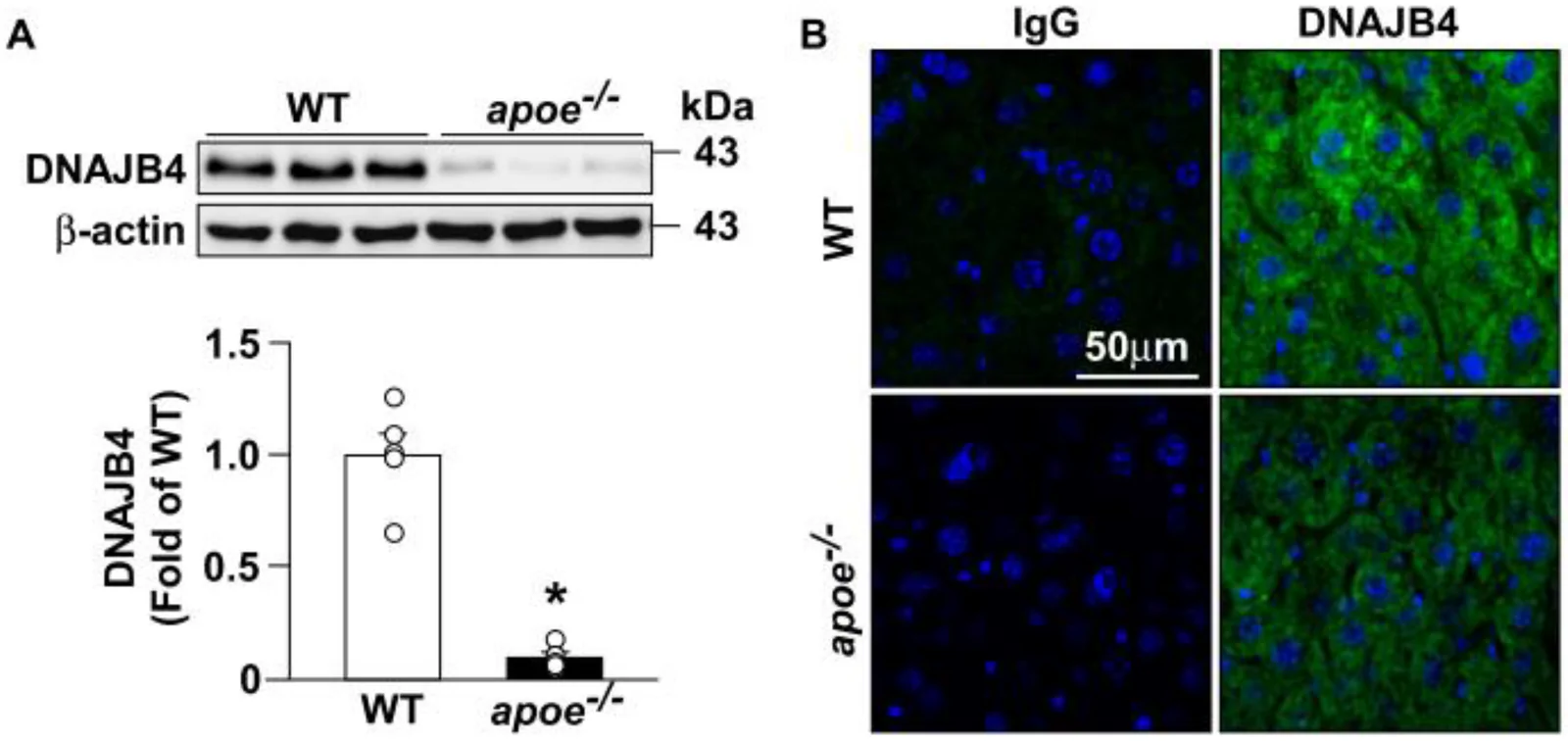
** DNAJB4 protein expression is decreased in the liver of *apoe^-/-^* mice.** Livers from five-month-old male wild-type (WT) C57BL/6 mice and *apoe*^-/-^ mice fed with a regular chow diet were extracted, lysed, or harvested for histological analysis. (A) Western blot analysis of DNAJB4 and β-actin in livers. (B) Immunohistochemistry of DNAJB4 (green) in the liver sections of five-month-old *apoe^-/-^* and WT mice. The nucleus was stained with DAPI. Data are expressed as mean ± SEM from five mice (n=5). *p < 0.05 vs. WT mice.

To study the possible role of DNAJB4 in the liver, livers from WT mice were used. Our results showed that DNAJB4 was expressed in various liver cell types, including hepatocytes, endothelial cells, and macrophages. Notably, DNAJB4 expression was also observed in the nuclei of various cell types (**Fig. 1A-C**). However, the protein level of DNAJB4 was markedly decreased in *apoe^-/-^* mice compared to WT mice (**Fig. 2A**). In addition, the expression of DNAJB4 in the nuclei of the liver was also decreased in *apoe^-/-^* mice (**Fig. 2B**). These findings suggest that DNAJB4 may play a significant role in the liver and may be associated with gene regulation.

### Genetic deletion of dnajb4 disrupts hepatic cholesterol metabolism in apoe^-/-^ mice

**Figure 3:**
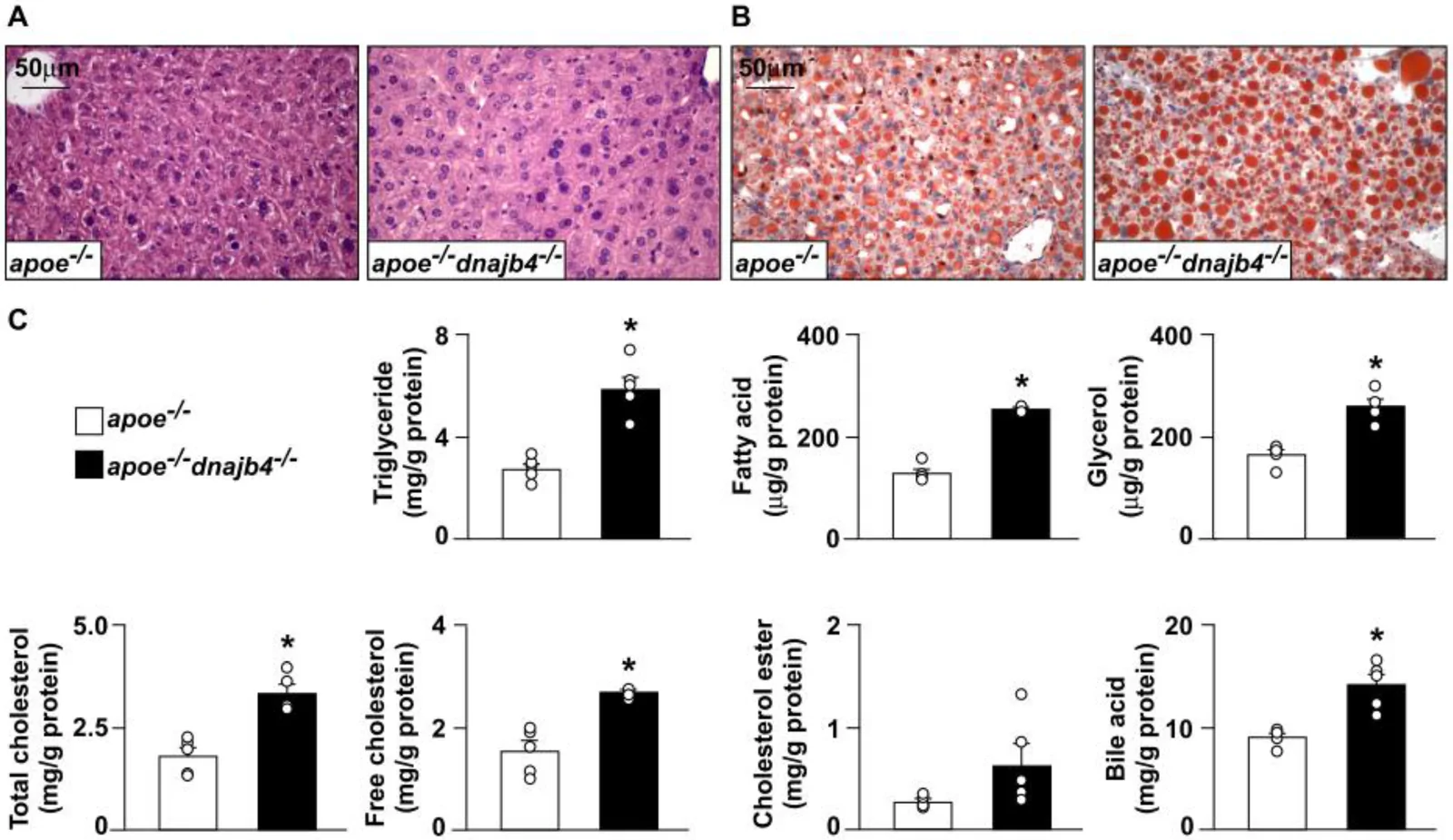
** Genetic deletion of *dnajb4* increases hepatic lipid accumulation in *apoe^-/-^* mice.** The *apoe*^-/-^*dnajb4*^-/-^ mice were generated by cross-breeding *apoe*^-/-^ and *dnajb4*^-/-^ mice. Livers from five-month-old male *apoe^-/-^* mice and *apoe^-/-^dnajb4^-/-^* mice fed with a regular chow diet were extracted for hepatic lipid analysis or harvested for histological analysis. (A and B) Representative histological images were obtained using (A) hematoxylin and eosin (H&E) staining and (B) oil red O staining. (C) The hepatic levels of triglyceride, fatty acid, glycerol, total cholesterol, free cholesterol, cholesterol ester, and bile acid. Data are expressed as mean ± SEM from five mice (n=5). *p < 0.05 vs. the *apoe*^-/-^ mice.

**Figure 4:**
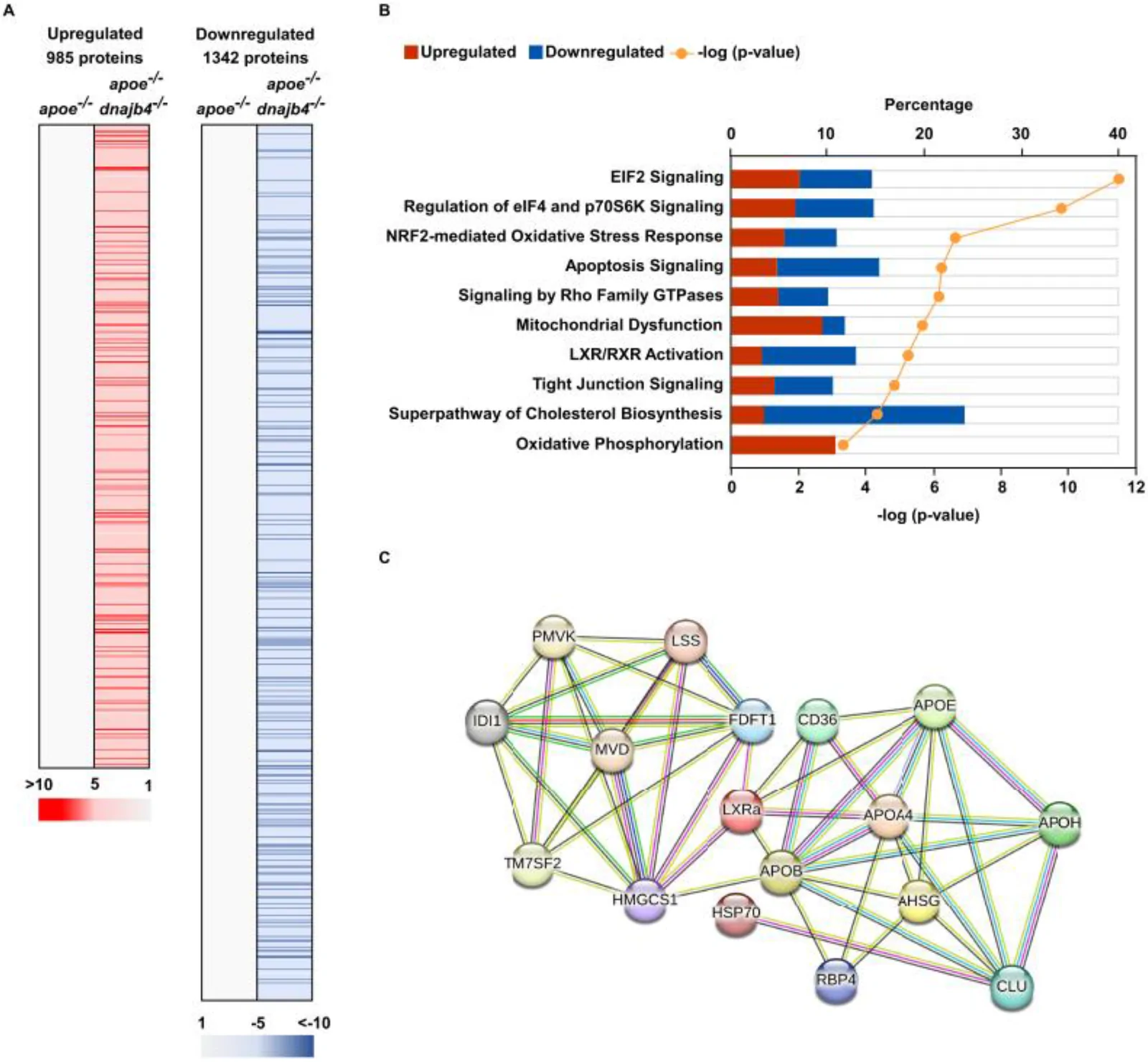
** Genetic deletion of *dnajb4* alters the protein expression in the livers of *apoe^-/-^* mice.** The *apoe*^-/-^*dnajb4*^-/-^ mice were generated by cross-breeding *apoe*^-/-^ and *dnajb4*^-/-^ mice. The livers were extracted from five-month-old male* apoe*^-/-^ and* apoe-/-dnajb4^-/-^* mice fed a regular chow diet and then lysed. (A) Proteomic analysis using LC-MS/MS identified altered proteins in the livers of *apoe*^-/-^*dnajb4*^-/-^ mice compared to *apoe*^-/-^ mice. (B) The ten selected canonical pathways related to lipid metabolism were identified using Ingenuity Pathway Analysis (IPA, 76765844M). Pathway significance was determined using -log(p-value), and pathways with -log(p-value) > 1.3 were considered statistically significant. (C) The protein-protein interaction (PPI) network was analyzed using the STRING database.

The liver is the primary organ responsible for lipid metabolism in humans. To investigate the potential role of DNAJB4 in hepatic lipid metabolism, we generated *apoe*^-/-^*dnajb4*^-/-^ mice. Compared with *apoe*^-/-^ mice, *apoe*^-/-^*dnajb4*^-/-^ mice showed more severe hepatic lipid accumulation (**Fig. 3A and B**). Furthermore, hepatic levels of triglyceride, fatty acid, glycerol, total cholesterol, free cholesterol, and bile acid were significantly increased in* apoe*^-/-^*dnajb4*^-/-^ mice compared with *apoe*^-/-^ mice (**Fig. 3C**). Liver samples analyzed using LC-MS/MS analysis revealed that 985 proteins were upregulated while 1342 proteins were downregulated in *apoe*^-/-^*dnajb4*^-/-^ mice **(Fig. 4A)**. Additionally, canonical pathway enrichment was performed using IPA, and pathways related to lipid metabolism were selected based on statistical significance **(Fig. 4B)**. To illustrate this in greater detail, **Tables 1** - **10** present the differentially expressed proteins associated with each of these pathways, as identified by IPA. These tables indicate proteins that were upregulated or downregulated (denoted by↑and↓, respectively), along with their biological functions and potential relevance to hepatic lipid metabolism. Lastly, the STRING database was utilized to construct a PPI network comprising the cholesterol metabolism-related targets in the canonical pathways, including LXRα and HSP70 **(Fig. 4C)**.

**Figure 5:**
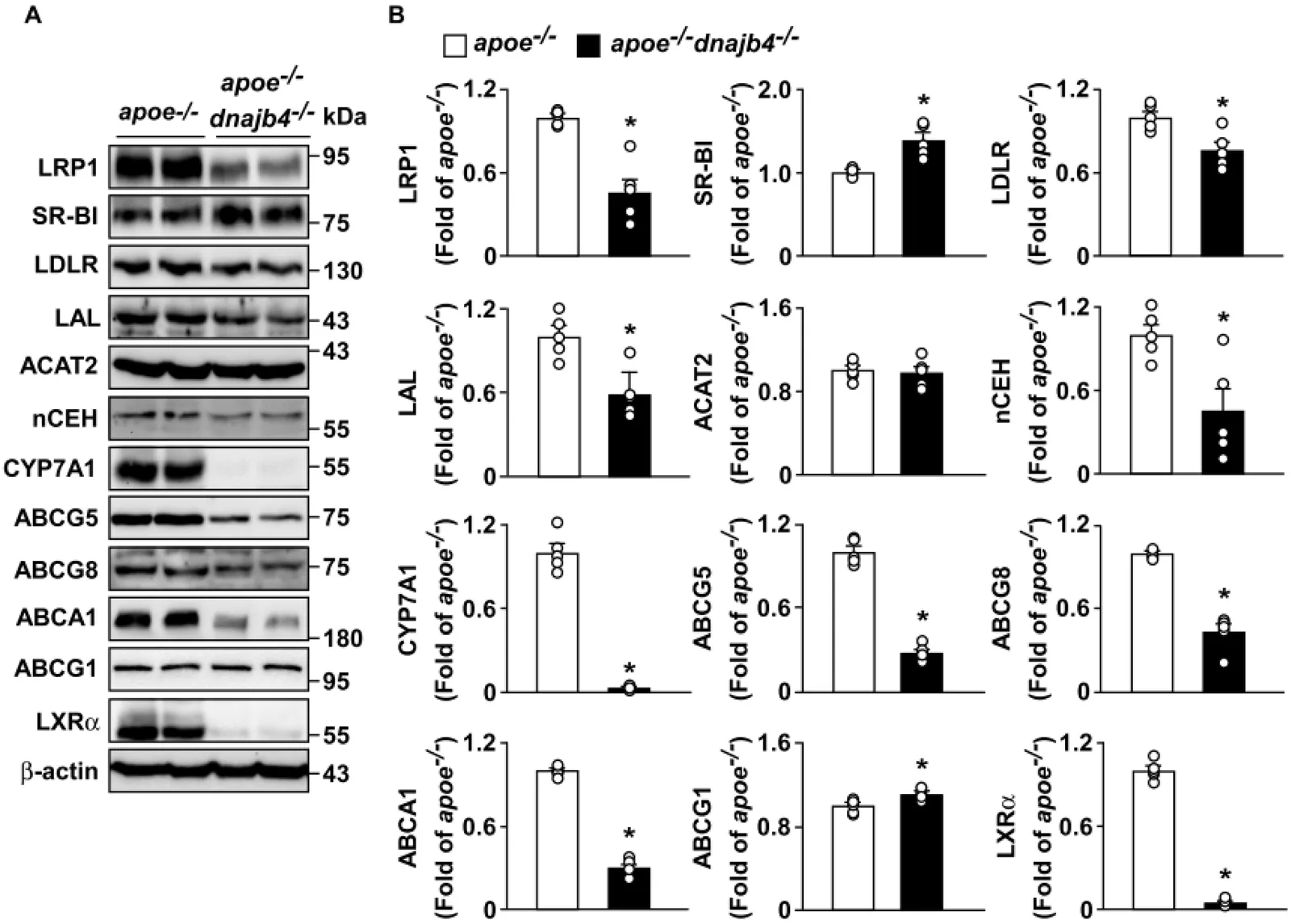
** Genetic deletion of *dnajb4* impairs hepatic cholesterol metabolism in *apoe^-/-^* mice.** The *apoe*^-/-^*dnajb4*^-/-^ mice were generated by cross-breeding *apoe*^-/-^ and *dnajb4*^-/-^ mice. The livers were extracted from five-month-old male* apoe*^-/-^ and apoe-/-dnajb4-/- mice fed a regular chow diet and then lysed. (A and B) Western blot analysis of LRP1, SR-B1, LDLR, LAL, ACAT2, nCEH, CYP7A1, ABCG5, ABCG8, ABCA1, ABCG1, LXRα, and β-actin in livers of *apoe^-/-^* mice and *apoe^-/-^dnajb4^-/-^* mice. Data are expressed as the mean ± SEM from five mice (n=5). *p < 0.05 vs. the *apoe*^-/-^ mice.

We further demonstrated that genetic deletion of *dnajb4* decreased protein expression of lipoprotein-uptake-related proteins, LRP-1 and LDLR, but increased SR-BI protein expression (**Fig. 5A and B**). Moreover, the expression of cholesterol esterification- and clearance-related proteins, including LAL, nCEH, CYP7A1, ABCG5, ABCG8, ABCA1, and LXRα, was decreased, but no significant changes in ACAT2 and ABCG1 in the liver of *apoe*^-/-^*dnajb4*^-/-^ mice (**Fig. 5A and B**). These results indicate that genetic deletion of *dnajb4* disrupts hepatic cholesterol esterification and clearance, leading to the accumulation of hepatic cholesterol.

### Genetic deletion of dnajb4 impairs HSP70-LXRα signaling in apoe^-/-^ mice

**Figure 6:**
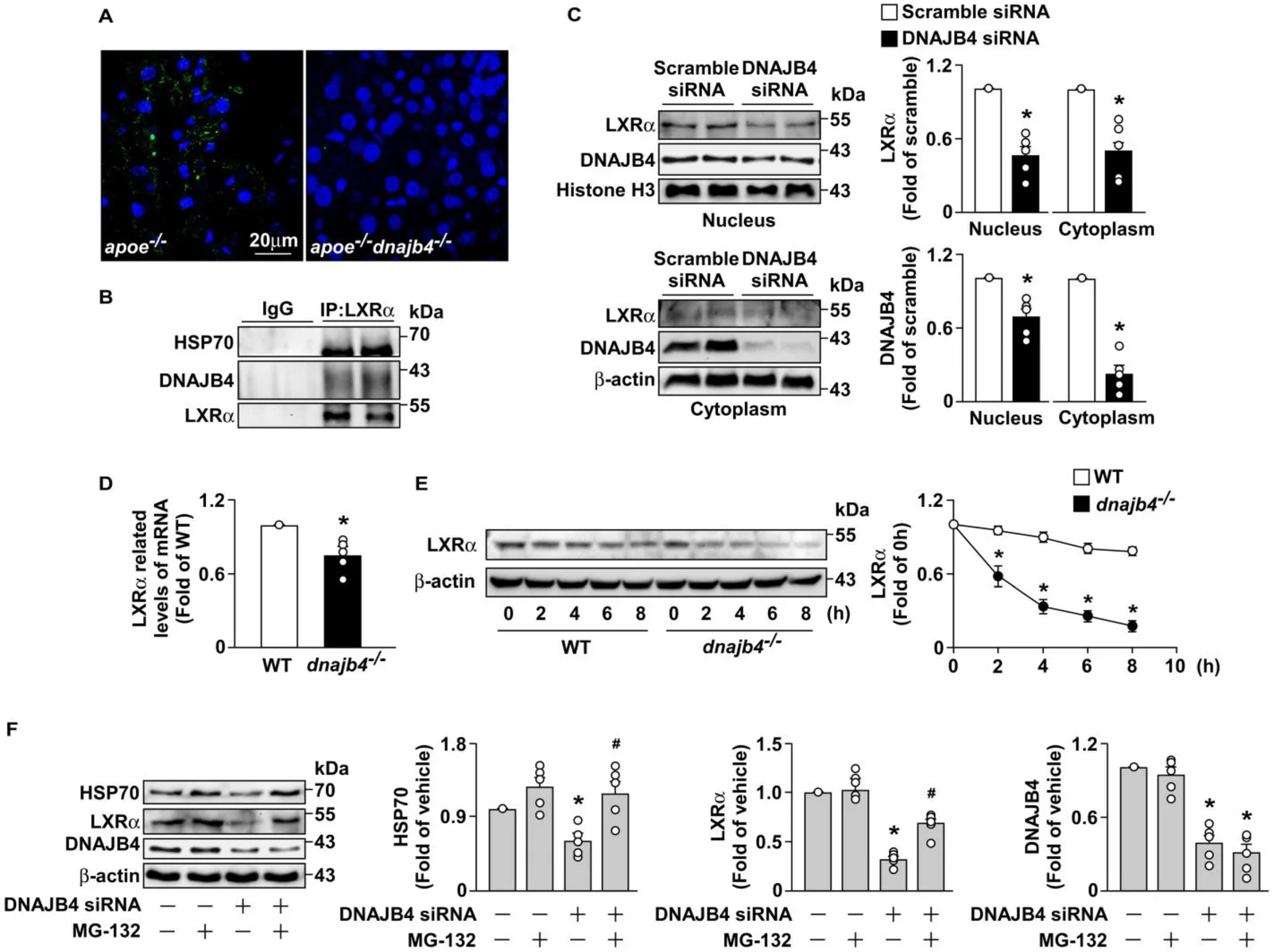
** The genetic deletion of *dnajb4* decreases LXRα gene expression and promotes LXRα protein degradation.** Livers and primary hepatocytes from five-month-old male wild-type (WT) C57BL/6 mice, *apoe^-/-^* mice, and *apoe^-/-^dnajb4^-/-^* mice fed a regular chow diet were isolated. (A) Representative confocal microscopy images of a proximity ligation assay (PLA) were obtained using anti-LXRα and anti-DNAJB4 antibodies. The nucleus was stained with DAPI, and PLA signals (green) were observed. (B) Co-immunoprecipitation assay of the interaction among HSP70, DNAJB4, and LXRα in the liver of WT mice. (C) Western blot analysis of LXRα and DNAJB4 in the nuclear and cytoplasmic fractions of Huh7 cells after transfection with scramble or DNAJB4 siRNA. (D) The mRNA expression of LXRα in the liver of WT and *dnajb4^-/-^* mice. (E) Western blot analysis of LXRα in primary hepatocytes isolated from WT and *dnajb4^-/-^* mice was performed after treatment with cycloheximide (20 μg/ml) at the indicated times (0, 2, 4, 6, and 8 h). (F) Western blot analysis of HSP70, LXRα, and DNAJB4 in Huh7 cells transfected with scramble or DNAJB4 siRNA, followed by treatment with or without proteasome inhibitor MG-132 (1 μM) for 12 h. Data are expressed as mean ± SEM from five mice (n=5). *p < 0.05 vs. the WT or scramble siRNA group. #*p* < 0.05 vs. the DNAJB4 siRNA group without MG-132 treatment.

**Figure 7:**
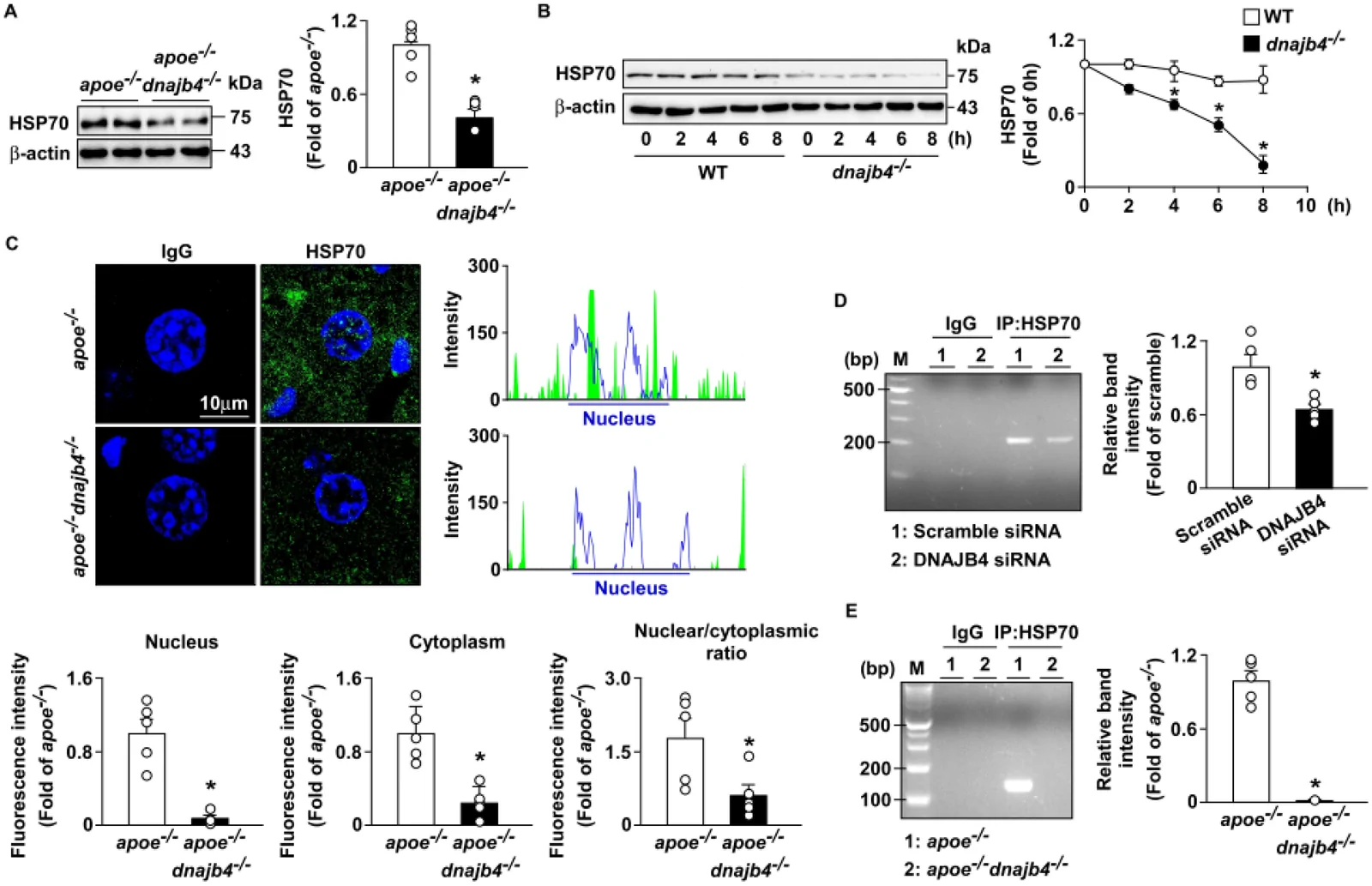
** Genetic deletion of *dnajb4* reduces nuclear HSP70 association and HSP70-regulated LXRα gene expression.** The livers or primary hepatocytes from five-month-old male WT mice, *dnajb4^-/-^* mice,* apoe^-/-^* mice, and *apoe^-/-^dnajb4^-/-^* mice fed with a regular chow diet were extracted or isolated. (A) Western blot analysis of HSP70 in the livers of *apoe^-/-^* mice and *apoe^-/-^dnajb4^-/-^* mice. (B) Western blot analysis of HSP70 in primary hepatocytes isolated from WT mice and *dnajb4^-/-^* mice were treated with cycloheximide (20 µg/ml) at the indicated times (0, 2, 4, 6, and 8 h). (C) Representative confocal microscopy images and quantified fluorescence intensity of HSP70 in the livers of *apoe^-/-^* mice and *apoe^-/-^dnajb4^-/-^* mice. The nuclear and cytoplasmic fluorescence intensities were quantified using ImageJ, and the nuclear regions were defined by DAPI staining. Chromatin immunoprecipitation (ChIP) with anti-HSP70 antibody followed by qRT-PCR of the LXRα promoter in (D) Huh7 cell line transfected with scramble siRNA or DNAJB4 siRNA and in (E) primary hepatocytes from *apoe^-/-^* mice and *apoe^-/-^dnajb4^-/-^* mice. Data are expressed as the mean ± SEM from five mice (n=5). *p < 0.05 vs. the WT or *apoe*^-/-^ mice.

Based on our findings in** Fig. 5**, the genetic deletion of *dnajb4* reduced the protein level of LXRα, the major transcription factor for genes involved in cholesterol metabolism. We further identified a potential molecular mechanism by which DNAJB4 regulates LXRα. We first investigated whether DNAJB4 physically associates with LXRα to exert its regulation. Results from PLA **(Fig. 6A)** and co-immunoprecipitation assays **(Fig. 6B)** provided definitive biochemical evidence of a physical interaction among DNAJB4, HSP70, and LXRα in the livers of WT mice. We next examined the subcellular distribution of LXRα to determine whether this reduction occurs. **Fig. 6C** revealed that the absence of DNAJB4 reduces LXRα protein levels in both the nuclear and cytoplasmic compartments. To determine whether this systemic reduction in LXRα was primarily due to decreased mRNA transcription or accelerated protein degradation, we evaluated both transcriptional levels and protein stability. Quantitative PCR analysis demonstrated that the mRNA level of LXRα was significantly decreased in *dnajb4^-/^*^-^ mice compared with WT mice (**Fig. 6D**). Moreover, genetic deletion of *dnajb4* promoted LXRα protein degradation in the primary hepatocytes compared with WT mice (**Fig. 6E**). Additionally, the decreased protein levels of LXRα, as well as HSP70, were rescued via the treatment of proteasome inhibitor MG-132, suggesting the accelerated protein clearance mediated by the proteasome pathway in the absence of DNAJB4. Collectively, these findings indicate that DNAJB4 physically interacts with LXRα and is essential for protecting from proteasomal degradation. As a co-chaperone of HSP70, we further investigated the molecular mechanism by which DNAJB4 regulates LXRα and HSP70. Our results showed that the protein level of HSP70 was significantly decreased in the liver of* apoe*^-/-^*dnajb4*^-/-^ mice compared with* apoe^-/-^* mice (**Fig. 7A**)*.* We then examined how the genetic deletion of *dnajb4* affected the degradation of HSP70 protein by CHX assay. Genetic deletion of *dnajb4* significantly promoted the HSP70 protein degradation (**Fig. 7B**). Moreover, confocal microscopy analysis revealed a significant decrease in the nuclear fluorescence intensity of HSP70 in the liver of *apoe*^-/-^*dnajb4*^-/-^ mice (**Fig. 7C**). According to a previous study, HSP70 interacted with the LXRα promoter and regulated the LXRα-related downstream gene expressions 18. We then further explored the crosstalk among DNAJB4, HSP70, and LXRα. Notably, our results showed that DNAJB4 knockdown reduced the occupancy of HSP70 at the LXRα promoter region in Huh7 cells; the same effect was observed in primary hepatocytes from *apoe*^-/-^*dnajb4*^-/-^ mice compared with *apoe*^-/-^ mice (**Fig. 7D and E**). These findings demonstrate that genetic deletion of dnajb4 reduces LXRα and HSP70 protein levels and decreases HSP70 protein stability and nuclear association, thereby inhibiting HSP70 binding to the LXRα promoter. This reduces LXRα-regulated downstream gene expression, ultimately leading to abnormal lipid metabolism in the mouse liver.

### Genetic deletion of dnajb4 eliminates the beneficial effect of curcumin and andrographolide on HSP70/LXRα-related hepatic cholesterol metabolism in apoe^-/-^ mice

**Figure 8:**
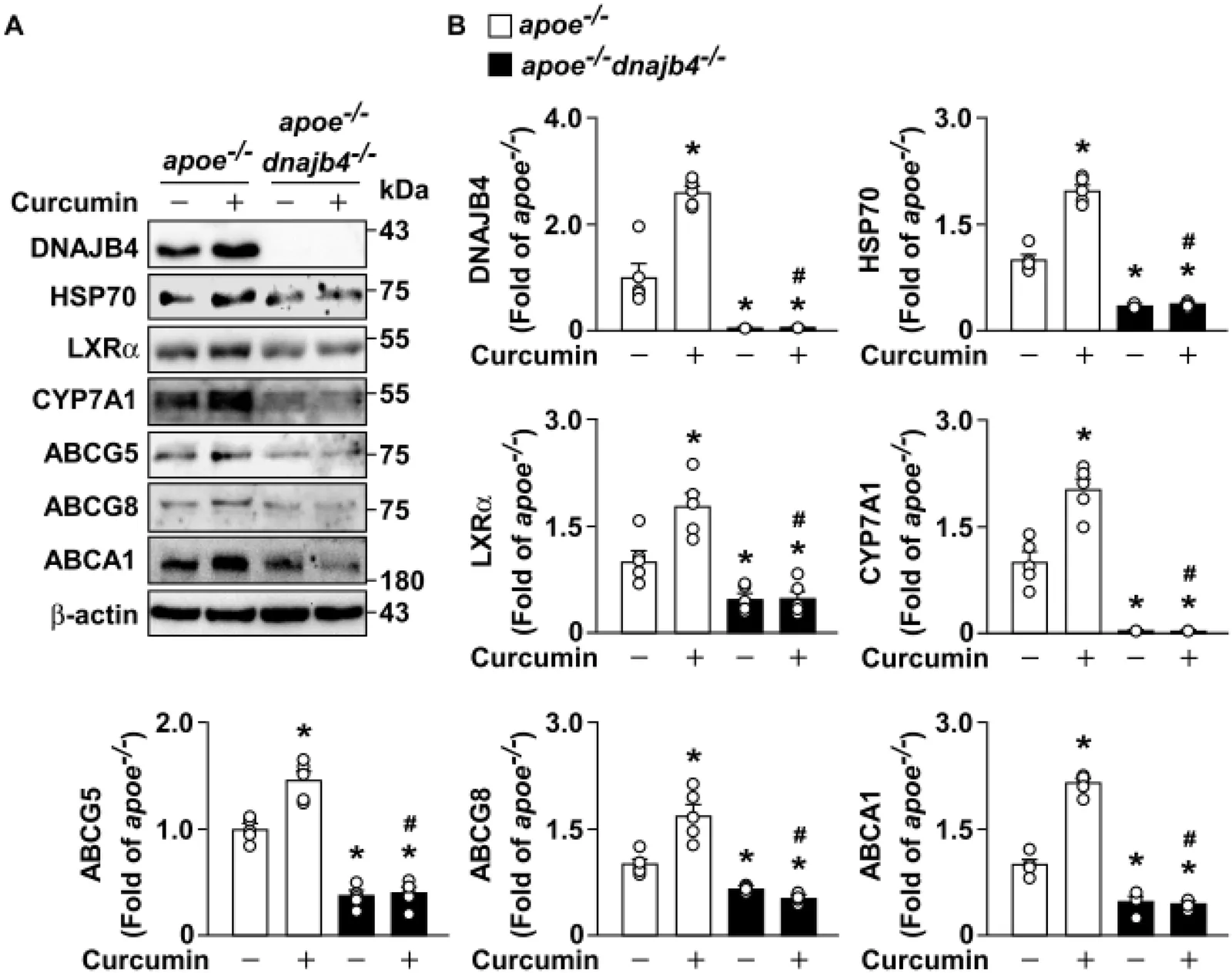
** DNAJB4 is required for the protective effect of curcumin in hepatic cholesterol metabolism in *apoe^-/-^* mice.** Four-month-old male *apoe^-/-^ mice* and *apoe^-/-^dnajb4^-/-^* mice fed with a regular chow diet were orally administered with curcumin (20 mg/kg) or vehicle (PBS) for four weeks. Mouse livers were extracted and lysed. (A and B) Western blot analysis of protein levels of DNAJB4, HSP70, LXRα, CYP7A1, ABCG5, ABCG8, ABCA1 and β-actin in the livers of *apoe^-/-^* mice and *apoe^-/-^dnajb4^-/-^* mice. Data are expressed as the mean ± SEM from five mice (n=5). * p < 0.05 vs. the vehicle group. ^#^* p* < 0.05 vs. the curcumin group in *apoe^-/-^* mice.

**Figure 9:**
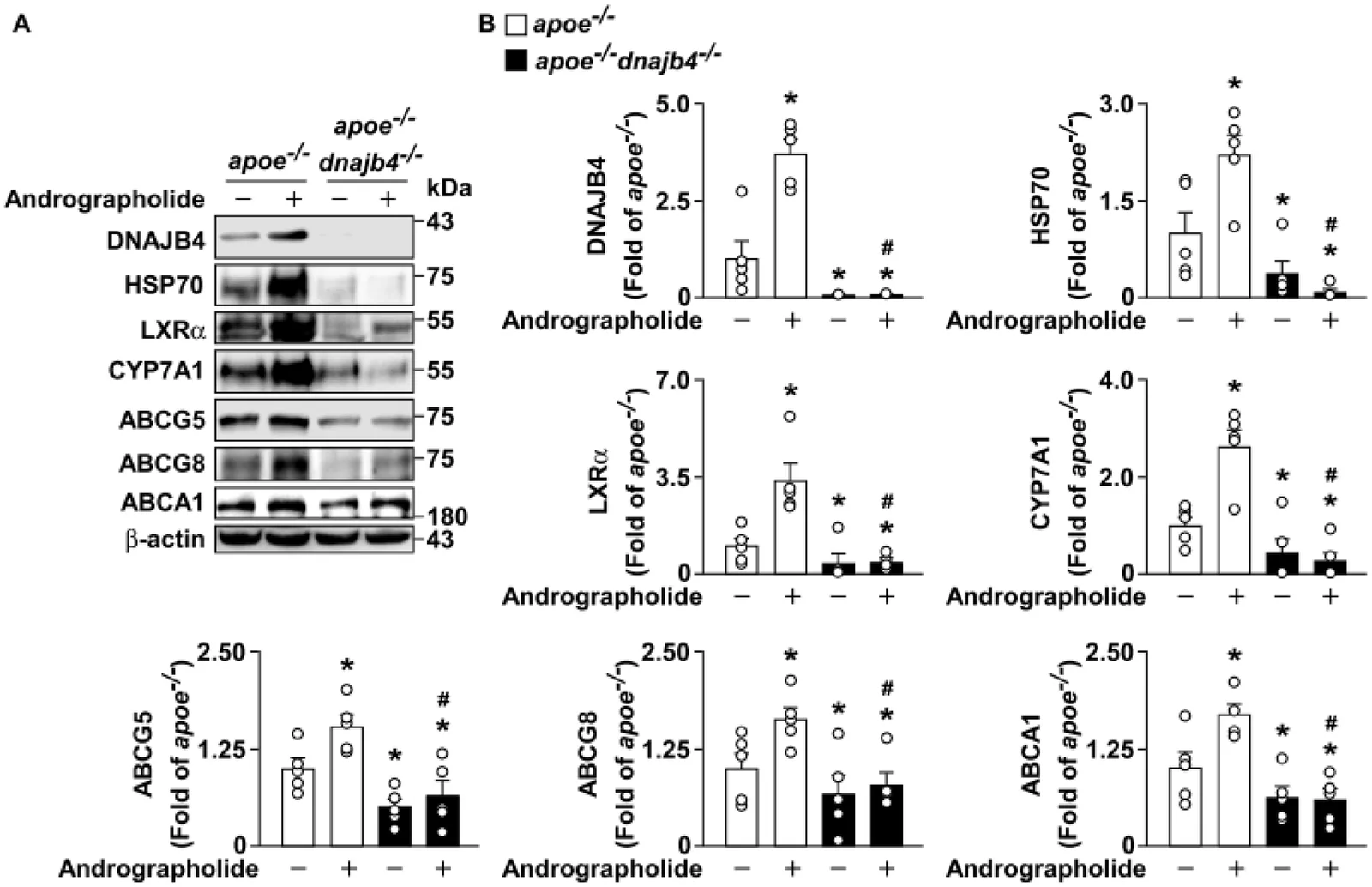
** DNAJB4 is required for the protective effect of andrographolide in hepatic cholesterol metabolism in *apoe^-/-^* mice.** Four-month-old male *apoe^-/-^* mice and *apoe^-/-^dnajb4^-/-^* mice fed with a regular chow diet were orally administered with andrographolide (20 mg/kg) or vehicle (PBS) for four weeks. Mouse livers were extracted and lysed. (A and B) Western blot analysis of DNAJB4, HSP70, LXRα, CYP7A1, ABCG5, ABCG8, ABCA1 and β-actin in the livers of *apoe^-/-^* mice and *apoe^-/-^dnajb4^-/-^* mice. Data are expressed as the mean ± SEM from five mice (n=5). *p < 0.05 vs. the vehicle group. ^#^*p* < 0.05 vs. the andrographolide group in *apoe^-/-^* mice.

To identify the role of DNAJB4 in hepatic cholesterol metabolism, *apoe*^-/-^ and* apoe*^-/-^*dnajb4*^-/-^ mice were treated with or without curcumin and andrographolide, which are inducers of DNAJB4 **19, 20**. Our data showed that DNAJB4, HSP70, and LXRα protein levels were noticeably increased in the liver of *apoe*^-/-^ mice treated with curcumin (**Fig. 8A and B**). Moreover, the LXRα-related streaming protein expressions were also significantly increased in the liver of *apoe*^-/-^ mice treated with the curcumin group, including CYP7A1, ABCG5, ABCG8, and ABCA1, compared with the vehicle group (**Fig. 8A and B**). However, the proteins upregulated by curcumin treatment in the liver of *apoe*^-/-^ mice could not be successfully induced or activated in the liver of *apoe*^-/-^*dnajb4*^-/-^ mice (**Fig. 8A and B**). Similar results were observed in *apoe*^-/-^ and *apoe*^-/-^*dnajb4*^-/-^ mice treated with or without andrographolide (**Fig. 9A and B**). These findings indicate that DNAJB4 is crucial for regulating hepatic cholesterol metabolism via the HSP70/LXRα pathway.

### Genetic deletion of dnajb4 abolished the beneficial effect of curcumin and andrographolide on hepatic lipid metabolism and against atherosclerosis in apoe^-/-^ mice

**Figure 10:**
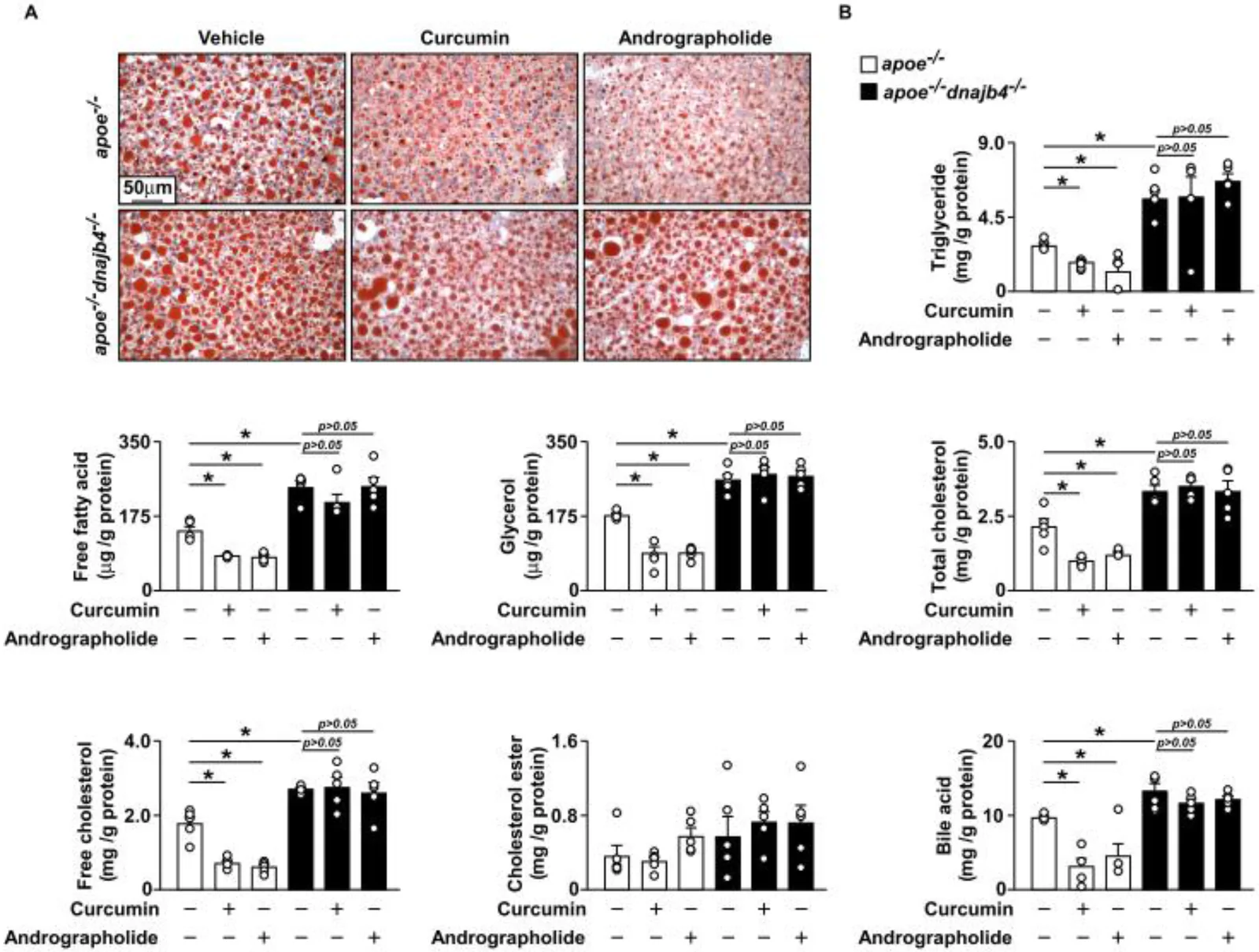
** The beneficial effects of curcumin or andrographolide in hepatic cholesterol accumulation in *apoe^-/-^* mice are partially dependent on DNAJB4.** Four-month-old male *apoe^-/-^* mice and *apoe^-/-^dnajb4^-/-^* mice fed with a regular chow diet were orally administered with curcumin (20 mg/kg), andrographolide (20 mg/kg), or vehicle (PBS) for four weeks. Mouse livers were extracted and lysed. (A) Representative histological images, as shown by Oil Red O staining, of the liver tissues. (B) The hepatic levels of triglyceride, fatty acid, glycerol, total cholesterol, free cholesterol, cholesterol ester, and bile acid. Data are expressed as mean ± SEM from five mice (n=5). *p < 0.05 vs. the vehicle group in *apoe^-/-^* mice.

**Figure 11:**
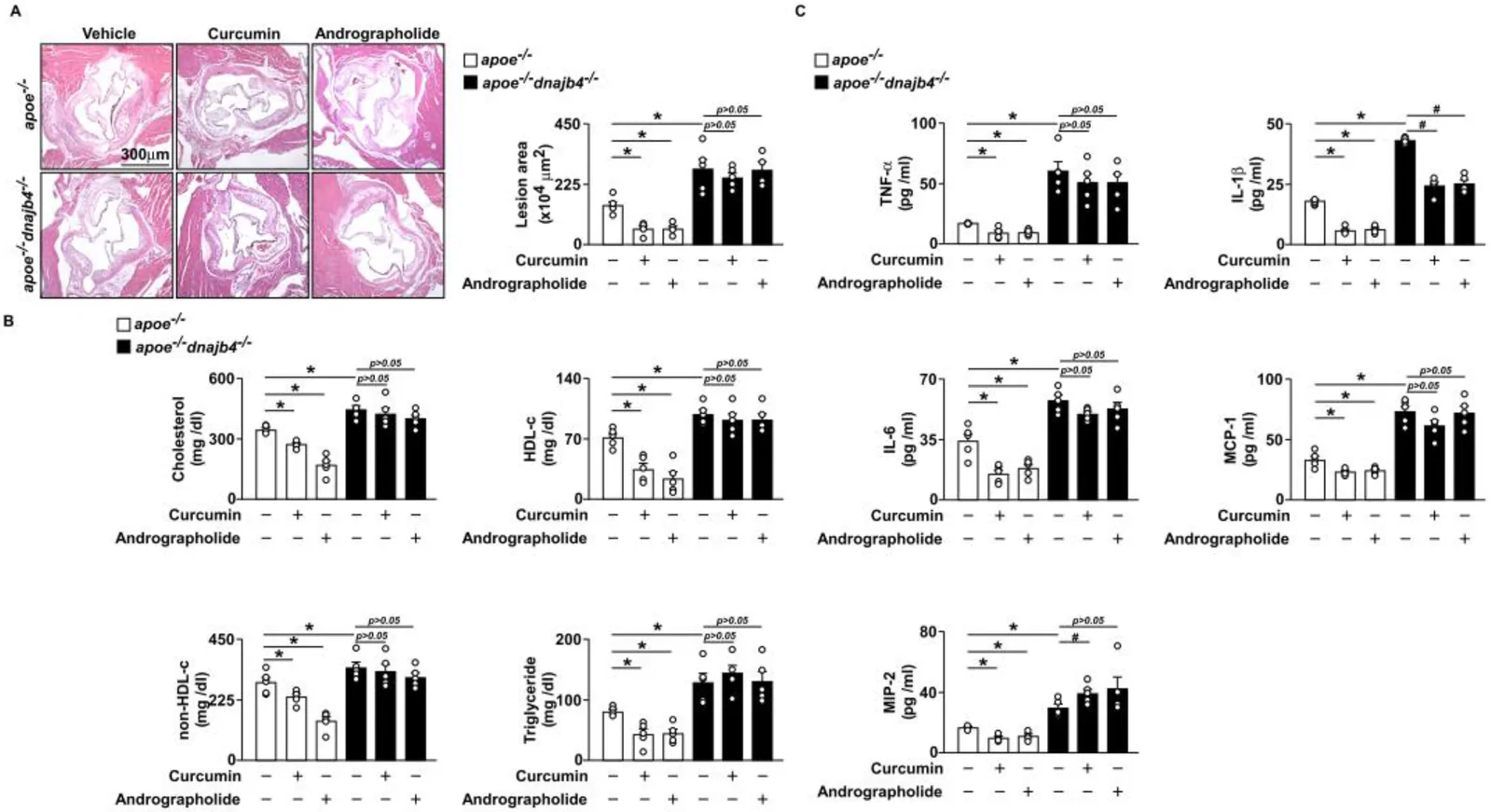
** The genetic deletion of *dnajb4* attenuated the protective effects of curcumin and andrographolide against atherosclerosis progression.** Four-month-old male *apoe^-/-^* mice and *apoe^-/-^dnajb4^-/-^* mice fed with a regular chow diet were orally administered with curcumin (20 mg/kg), andrographolide (20 mg/kg), or vehicle (PBS) for four weeks. The hearts and plasma of the mice were collected. (A) Representative images and quantifications of atherosclerotic lesions at the aortic roots were obtained after H&E staining and analysis. (B) Plasma levels of cholesterol, high-density lipoprotein cholesterol (HDL-c), non-HDL-c, triglyceride, TNF-α, IL-1β, IL-6, MCP-1, and MIP-2. Data are expressed as mean ± SEM from four to five mice (n=4-5). * p < 0.05 vs. the vehicle group in *apoe^-/-^* mice. ^#^* p* < 0.05 vs. the vehicle group in *apoe^-/-^dnajb4^-/-^* mice.

Abnormal hepatic cholesterol metabolism may lead to hyperlipidemia and hyperlipidemia-induced atherosclerosis 22. To further elucidate the role of DNAJB4 in hepatic cholesterol metabolism and atherosclerosis, we next investigated its contribution to hepatic lipid accumulation. Our results determined that the hepatic lipid accumulation and the hepatic level of triglyceride, fatty acid, glycerol, total cholesterol, free cholesterol, and bile acid were significantly reduced after *apoe*^-/-^ mice treated with curcumin and andrographolide; however, the protective effect of curcumin and andrographolide on hepatic lipid accumulation was abolished in *apoe*^-/-^*dnajb4*^-/-^ mice (**Fig. 10A and B**). We then examined the role of DNAJB4 in atherosclerotic lesions. According to our findings, the atherosclerotic lesions and plasma lipid levels of cholesterol, HDL-c, non-HDL-c, and triglyceride were significantly decreased in *apoe*^-/-^ mice treated with curcumin and andrographolide; however, the protective effect of curcumin and andrographolide against hyperlipidemia and atherosclerotic lesions was eradicated in *apoe*^-/-^*dnajb4*^-/-^ mice (**Fig. 11A and B**). Moreover, the plasma inflammation cytokines levels including TNF-α, IL-1β, IL-6, MCP-1, and MIP-2 were also significantly decreased in *apoe*^-/-^ mice treated with curcumin and andrographolide; however, the beneficial effect of curcumin and andrographolide against inflammation was eliminated in *apoe*^-/-^*dnajb4*^-/-^ mice (**Fig. 11C**). Taken together, these findings suggest that DNAJB4 plays a crucial role in the regulation of hepatic lipid metabolism and atherosclerosis.

## Discussion

**Figure 12:**
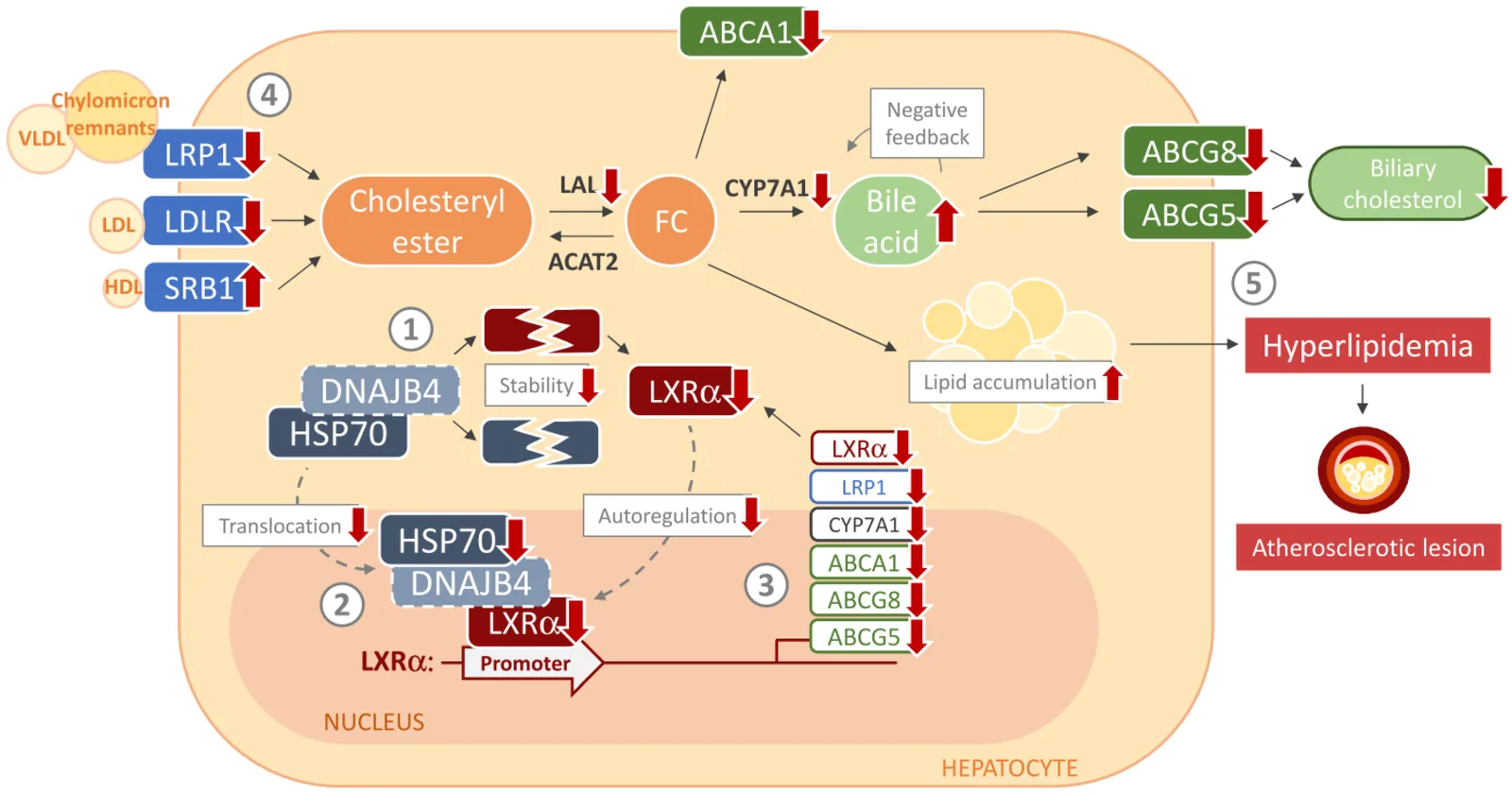
** Schematic illustration of the proposed molecular mechanisms by which the genetic deletion of DNAJB4 disturbs hepatic lipid metabolism and exacerbates hyperlipidemia-induced atherosclerosis by impairing HSP70-LXRα signaling.** In the context of *dnajb4* deficiency, the sequence of events is illustrated as follows: (1) The disruption of the DNAJB4-HSP70 leads to decreased protein stability of HSP70 and LXRα. (2) This results in reduced nuclear HSP70 association and diminished binding of HSP70 to the LXRα promoter. (3) Consequently, the transcription of LXRα and its downstream target genes involved in cholesterol metabolism is significantly downregulated. (4) These transcriptional alterations disrupt hepatic cholesterol homeostasis, leading to altered lipoprotein receptor expression and impaired cholesterol clearance. (5) Ultimately, this cascade of molecular dysregulation results in the aggravation of hepatic lipid accumulation, hyperlipidemia, and the progression of atherosclerotic lesions.

In this study, we characterized a novel function of DNAJB4 and its potential molecular mechanism in hepatic cholesterol metabolism and atherosclerosis. Our findings suggested that genetic deletion of DNAJB4 promotes protein degradation of HSP70 and LXRα, ultimately deregulating hepatic cholesterol metabolism and exacerbating atherosclerosis progression (**Fig. 12**). Our results demonstrated that DNAJB4 is localized to hepatocytes and endothelial cells in the livers of WT mice. Furthermore, in the atherosclerotic lesions of *apoe^-/-^* mice, its expression was observed in both endothelial cells and foam cells (**Fig. S1**). Moreover, siRNA-mediated knockdown of DNAJB4 decreased nitrite production in endothelial cells **(Fig. S2)** and increased the accumulation of oxidized LDL compared with the scramble siRNA group (**Fig. S3**). Therefore, *apoe^-/-^dnajb4^-/-^* mice were used to investigate the role of DNAJB4 in hepatic cholesterol metabolism and atherosclerosis. Mechanistically, genetic deletion of *dnajb4* promoted the degradation of LXRα and HSP70 proteins and reduced nuclear association of HSP70, thereby decreasing HSP70 binding to the LXRα promoter and lowering expression of LXRα-downstream cholesterol-related proteins. Importantly, LXRα functions as an autoregulatory transcription factor; its initial protein degradation not only represses the transcription of downstream cholesterol-related genes but also blunts its own transcription **17**. Consequently, this feed-forward suppression exacerbates hepatic lipid accumulation and atherosclerosis in *apoe^-/-^dnajb4^-/-^* mice. These findings suggest that the genetic deletion of *dnajb4* disrupts HSP70/LXRα crosstalk, worsens hepatic cholesterol metabolism, causes hepatic accumulation, and ultimately increases hyperlipidemia and the development of atherosclerotic lesions.

Our findings in Fig.1 show that DNAJB4 is expressed in hepatocytes, endothelial cells, and macrophages. Hepatic endothelial cells are central to lipid exchange with hepatocytes in hepatic lipid metabolism. Endothelial cell dysfunction leads to immune cell infiltration into the liver, thereby contributing to metabolic diseases 23. Moreover, dysregulation of Kupffer cells, the resident macrophages of the liver, can also drive immune cell infiltration and subsequent metabolic disease 24, 25. Additionally, endothelial cell dysfunction and the abnormal formation of macrophage foam cells also play critical roles in the development of atherosclerosis 26, 27. Evaluation of DNAJB4 distribution in the aorta revealed that it is predominantly expressed in the endothelial cells of WT mice. At the same time, its expression profile broadens to include both endothelial cells and foam cells within the atherosclerotic lesions of *apoe^-/-^* mice **(Fig. S1)**. Notably, DNAJB4 protein expression is distinctly absent in smooth muscle cells. These expression patterns suggest that DNAJB4 may play a regulatory role during the early stages of atherosclerosis. However, whether DNAJB4 also plays a vital role in lipid metabolism in endothelial cells and macrophages remains unclear. Furthermore, Panjwani *et al.* reported that, with progressive atherosclerosis, HSP70 levels decrease due to reduced nitric oxide release into the circulation, resulting from endothelial dysfunction 28. HSP70 broadly regulates cellular hemostasis, including inflammation, oxidative stress, and the unfolded protein response 29-31. Liu *et al.* and Bielecka-Dabrowa et al. showed that the induction of HSP70 protected vascular smooth muscle cells from oxidized-LDL-induced apoptosis 32, 33. However, whether the protective effects of HSP70 against atherosclerosis in endothelial cells and macrophage foam cells are mediated by DNAJB4 remains to be investigated. Although the present study incorporated mechanistic experiments using human microvascular endothelial cells and macrophage foam cells, validation in human atherosclerotic arterial specimens will be important for confirming whether the DNAJB4-HSP70-LXRα axis identified in our experimental models is similarly dysregulated in human vascular disease.

Although the present study employed a global *dnajb4* knockout model, this approach was appropriate for determining the overall physiological contribution of DNAJB4 to systemic lipid homeostasis and atherosclerosis in the hyperlipidemic *apoe^-/-^* background. The marked alterations in hepatic cholesterol metabolism, together with accelerated vascular lesion formation, support an important role for DNAJB4 in regulating the HSP70-LXRα axis *in vivo*. Nevertheless, because DNAJB4 is expressed in hepatocytes, endothelial cells, and macrophages, the current model cannot distinguish the relative contribution of each cell population to the observed phenotype. Future studies using hepatocyte-, endothelial cell-, or macrophage-specific *dnajb4* knockout models will therefore be valuable for defining the tissue- and cell-specific functions of DNAJB4 during atherosclerosis progression. Collectively, these findings support hepatic dysregulation of the DNAJB4-HSP70-LXRα axis as an important upstream mechanism linking DNAJB4 deficiency to systemic hyperlipidemia and subsequent atherosclerotic lesion development. Although additional effects of DNAJB4 deficiency in vascular and immune cells cannot be excluded, the present study was primarily designed to elucidate its role in hepatic cholesterol metabolism.

In our study, genetic deletion of *dnajb4* in *apoe*^-/-^ mice results in increased LXRα protein degradation in the liver, leading to decreased LXRα protein expression. Importantly, because LXRα functions as an autoregulatory transcription factor, this initial protein degradation further blunts its own gene transcription. The exacerbated lipid accumulation in *apoe^-/-^dnajb4^-/-^* mice with reduced LXRα protein expression is consistent with Peet et al., who reported that *lxrα*^-/-^ mice exhibit hepatic cholesterol accumulation due to impaired CYP7A1 expression 34. Our results align with the findings of Tangirala *et al*., who demonstrated that loss of LXRα function accelerates the initiation and progression of atherosclerosis in *apoe^-/-^* mice 13. Beyond atherosclerosis, LXRα is also associated with other metabolic diseases. Laffitte *et al.* demonstrated that administering the LXR agonist GW3965 to diet-induced obese mice significantly improved glucose tolerance in the liver and adipose tissues 35. Moreover, Zhou *et al*. reported that LXRα activation ameliorated diet-induced hepatic steatosis and inflammation in mice treated with SH42, a Δ24-dehydrocholesterol reductase inhibitor 36. Although LXRα plays a critical role in modulating these metabolic diseases, further research is needed to elucidate the relationship between DNAJB4-mediated regulation of LXRα and other metabolic disorders.

Furthermore, our fractionation data revealed that the loss of DNAJB4 leads to a significant decrease in LXRα protein levels in both the cytoplasmic and nuclear compartments. As a co-chaperone of the HSP70 machinery, DNAJB4 is critical for the proper folding and stabilization of newly synthesized proteins. In the absence of DNAJB4, misfolded LXRα proteins in the cytoplasm may be degraded via the proteasome prior to nuclear translocation. A previous study has also demonstrated that *de novo* synthesized proteins are highly vulnerable to proteasomal clearance without the immediate surveillance of the chaperone network 38. Importantly, the degradation dynamics of transcription factors and nuclear receptors are highly dependent on their subcellular localization. Mature nuclear receptors, including the estrogen receptor and the glucocorticoid receptor, when anchored to chromatin complexes, typically exhibit prolonged half-lives due to structural protection against ubiquitination 39, 40. Thus, while the existing nuclear pool of LXRα initially remains stable, its natural turnover combined with a complete lack of newly synthesized replacements from the cytoplasm ultimately results in the eventual exhaustion of nuclear LXRα. These findings suggested that DNAJB4 is essential for *de novo* protein synthesis and stabilizes LXR in the cytoplasm before its nuclear entry. Beyond these proteasome-dependent effects on protein stability, accumulating evidence suggests that DNAJ/HSP40 family members, including DNAJB4, possess biological activities, such as participation in protein trafficking and transcription-associated processes 2, 41. This raises the intriguing possibility that DNAJB4 also exerts protein stabilization-independent functions within the nucleus. Consequently, the reduced nuclear LXRα activity observed in our study may not result exclusively from the accelerated proteasomal degradation. DNAJB4 could facilitate the assembly of transcriptionally competent LXR complexes, regulate their dynamic interactions with RXR, chromatin, and co-regulators, or contribute to the nuclear retention and intranuclear trafficking of LXRα 2, 39, 40. While our current findings primarily establish a proteasome-dependent degradation mechanism, they provide a conceptual framework for the multifaceted roles of DNAJB4. Future studies employing techniques such as chromatin immunoprecipitation and live-cell imaging are warranted to determine whether DNAJB4 also functions as a multifunctional regulator that directly orchestrates the LXRα transcriptional machinery.

Notably, in our study, we found that DNAJB4 participates in the HSP70*-*LXRα cross-talk. This finding is consistent with Gungor *et al.* who demonstrated that HSP70 interacted with the macrophage LXRα promoter and enhanced LXRα-targeted gene expression, thereby facilitating cholesterol efflux 18. However, it is worth exploring whether the protein-protein interaction between DNAJB4 and HSP70 occurs before or after nuclear translocation. All Hsp70 chaperone activities require the regulatory and substrate-targeting functions of co-chaperones in the J-domain protein family, such as DNAJB4 37. In the cytosol, HSP70 and HSP40, like DNAJB4, regulate protein disaggregation, refolding, and degradation 42-44. Therefore, the cytosolic protein-protein interactions between HSP70 and DNAJB4 may contribute to LXRα stability. Furthermore, Li et al. suggested that HSP70 can enter the nucleus via the nuclear import carrier Hikeshi and promote murine oligodendrocyte differentiation and central nervous system myelination 45. Previous studies have indicated that the Hsp40 proteins Ydj1 and Sis1 are required for nuclear import of substrates and can form an Hsp40/70 adaptor complex with the Hsp70 proteins Ssa1/Ssa2 to deliver misfolded proteins into the nucleus for quality control 46. These findings demonstrate that HSP40 and HSP70 can enter the nucleus and form a complex that regulates protein levels. Therefore, the protein-protein interaction between DNAJB4 and HSP70 in the nucleus, as studied in this paper, can regulate LXRα binding to its promoter. However, the detailed molecular mechanisms underlying the regulation of this requirement by the DNAJB4-HSP70 interaction require further investigation and validation.

Despite our novel findings regarding the molecular mechanism of DNAJB4 in hepatic lipid accumulation and atherosclerosis, our study has several limitations. First, the present study employed a global* dnajb4* knockout model in the* apoe*^-/-^ background and therefore cannot distinguish the relative contributions of DNAJB4 in hepatocytes, endothelial cells, macrophages, and other cell populations involved in lipid metabolism and atherogenesis. Nevertheless, this model was suitable for establishing the overall physiological contribution of DNAJB4 to hepatic cholesterol dysregulation, hyperlipidemia, and atherosclerosis under hyperlipidemic conditions. We also did not comprehensively characterize the basal hepatic and metabolic phenotypes of single *dnajb4*^-/-^ mice. Therefore, future studies using single *dnajb4*^-/-^ mice and tissue-specific conditional knockout models, including hepatocyte-, endothelial cell-, and macrophage-specific deletion, will be required to define the basal, tissue-specific, and cell-specific functions of DNAJB4. Second, we did not provide direct evidence of an interaction between DNAJB4 and HSP70 in the nucleus. Future studies could employ nuclear import inhibitors to investigate whether the DNAJB4-HSP70 interaction occurs before or after nuclear translocation, thereby determining if DNAJB4 is strictly required for the nuclear entry of HSP70. Third, a technical limitation regarding hepatic cholesteryl ester quantification exists. Despite a logical expectation of cholesterol ester accumulation due to reduced nCEH and LAL, we observed only a trend toward an increase (*p*=0.08). This lack of statistical significance was likely driven by our relatively small sample size and the compounded analytical variance of indirectly deriving cholesterol ester from total and free cholesterol assays. Future studies utilizing larger animal cohorts or direct examination of lipid profiles are warranted. Lastly, our study lacks direct validation using human atherosclerotic arterial tissues and clinically characterized cardiovascular specimens. Such validation would further strengthen the translational relevance of the DNAJB4-HSP70-LXRα pathway identified in the present study. This limitation primarily arises from the distinct metabolic drivers represented in currently available public datasets, which predominantly involve morbid obesity or advanced NASH rather than hyperlipidemia-driven early hepatic lipid accumulation, as well as the ethical constraints associated with obtaining liver biopsies or vascular specimens from patients at early disease stages. Despite the absence of human tissue validation, we attempted to enhance the translational significance of this work by demonstrating that two naturally occurring DNAJB4 inducers, curcumin and andrographolide, effectively ameliorated hepatic cholesterol dysregulation and atherosclerosis *in vivo* in a DNAJB4-dependent manner. These findings provide a mechanistic rationale for future studies evaluating DNAJB4 expression in human atherosclerotic arteries and for prospective clinical investigations targeting the DNAJB4-HSP70-LXRα pathway.

In conclusion, this study demonstrates the new molecular mechanisms of DNAJB4 on hepatic lipid metabolism and atherosclerosis, which involves the regulation of LXRα and HSP70 protein stability, the protein-protein interaction of DNAJB4-HSP70, the increase in nuclear HSP70 association, the binding of HSP70 protein and LXRα promoter, transcription of LXRα-related cholesterol metabolism, and ultimately leads to aggravation of hepatic lipid accumulation, hyperlipidemia, and atherosclerosis (**Fig. 12**). Here we provide new insight into the role of DNAJB4 on hepatic cholesterol metabolism, which broadens the more possibilities for future treatment of atherosclerosis and related metabolic diseases.

## Supplementary Material

Supplementary figures and tables.

## Figures and Tables

**Table 1:** The effect of the genetic deletion of *dnajb4* in the livers of *apoe^-/-^* mice on EIF2 signaling pathway-related proteins.

Protein	Protein description	*apoe^-/-^dnajb4^-/-^* vs. *apoe^-/-^*	Fold change
ACTC1	Actin, alpha cardiac muscle 1	↑	2.62
EIF2B1	Translation initiation factor eIF-2B subunit alpha	↓	-3.68
EIF3B	Eukaryotic translation initiation factor 3 subunit B	↓	-3.94
EIF3G	Eukaryotic translation initiation factor 3 subunit G	↓	-1E+10
EIF3L	Eukaryotic translation initiation factor 3 subunit L	↑	2.06
EIF4A3	Eukaryotic initiation factor 4A-III	↓	-5.68
FAU	40S ribosomal protein S30	↓	-1E+10
GRB2	Growth factor receptor-bound protein 2	↓	-2.47
KRAS	GTPase KRas	↑	2
MAP2K2	Dual specificity mitogen-activated protein kinase kinase 2	↓	-3.7
MAPK1	Mitogen-activated protein kinase 1	↓	-2.95
PAIP1	Polyadenylate-binding protein-interacting protein 1	↓	-5.06
Ppp1cc	Serine/threonine-protein phosphatase PP1-gamma catalytic subunit	↓	-1E+10
RALA	Ras-related protein Ral-A	↑	2.03
RAP1A	Ras-related protein Rap-1A	↑	2.68
RAP2B	Ras-related protein Rap-2b	↓	-1E+10
RPL28	60S ribosomal protein L28	↑	30.28
RPL31	60S ribosomal protein L31	↑	3.6
RPL36a	60S ribosomal protein L36a	↓	-5.21
RPL38	60S ribosomal protein L38	↑	2.28
RPL5	60S ribosomal protein L5	↓	-3.89
RPL8	60S ribosomal protein L8	↑	2.27
RPS11	40S ribosomal protein S11	↑	2.58
RPS12	40S ribosomal protein S12	↑	2.32
RPS20	40S ribosomal protein S20	↓	-2.04
RPS24	40S ribosomal protein S24	↑	3.36
RPS26	40S ribosomal protein S26	↑	5.96
RPS5	40S ribosomal protein S5	↑	4.92
RPS7	40S ribosomal protein S7	↑	2.34
RRAS2	Ras-related protein R-Ras2	↓	-1E+10

**Table 2:** The effect of the genetic deletion of *dnajb4* in the livers of *apoe^-/-^* mice on the regulation of eIF4 and p70S6K signaling pathway-related proteins.

Protein	Protein description	*apoe^-/-^dnajb4^-/-^* vs. *apoe^-/-^*	Fold change
EIF2B1	Translation initiation factor eIF-2B subunit alpha	↓	-3.68
EIF3B	Eukaryotic translation initiation factor 3 subunit B	↓	-3.94
EIF3G	Eukaryotic translation initiation factor 3 subunit G	↓	-1E+10
EIF3L	Eukaryotic translation initiation factor 3 subunit L	↑	2.06
EIF4A3	Eukaryotic initiation factor 4A-III	↓	-5.68
FAU	40S ribosomal protein S30	↓	-1E+10
GRB2	Growth factor receptor-bound protein 2	↓	-2.47
ITGA9	Integrin alpha-9	↓	-2.81
ITGAV	Integrin alpha-V	↓	-31.66
ITGB3	Integrin beta-3	↓	-4.49
KRAS	GTPase KRas	↑	2
MAP2K2	Dual specificity mitogen-activated protein kinase kinase 2	↓	-3.7
MAPK1	Mitogen-activated protein kinase 1	↓	-2.95
PAIP1	Polyadenylate-binding protein-interacting protein 1	↓	-5.06
PPP2R2A	Serine/threonine-protein phosphatase 2A 55 kDa regulatory subunit B alpha isoform	↑	3.39
PPP2R5A	Serine/threonine-protein phosphatase 2A 56 kDa regulatory subunit alpha isoform	↑	2.65
RALA	Ras-related protein Ral-A	↑	2.03
RAP1A	Ras-related protein Rap-1A	↑	2.68
RAP2B	Ras-related protein Rap-2b	↓	-1E+10
RPS11	40S ribosomal protein S11	↑	2.58
RPS12	40S ribosomal protein S12	↑	2.32
RPS20	40S ribosomal protein S20	↓	-2.04
RPS24	40S ribosomal protein S24	↑	3.36
RPS26	40S ribosomal protein S26	↑	5.96
RPS5	40S ribosomal protein S5	↑	4.92
RPS7	40S ribosomal protein S7	↑	2.34
RRAS2	Ras-related protein R-Ras2	↓	-1E+10

**Table 3:** The effect of the genetic deletion of *dnajb4* in the livers of *apoe^-/-^* mice on NRF2-mediated oxidative stress response pathway-related proteins.

Protein	Protein description	*apoe^-/-^dnajb4^-/-^* vs. *apoe^-/-^*	Fold change
ABCC2	Canalicular multispecific organic anion transporter 1	↑	1E+10
ACTC1	Actin, alpha cardiac muscle 1	↑	2.62
ACTG1	Actin, cytoplasmic 2	↑	3.55
AOX1	Aldehyde oxidase 1	↑	2.06
DNAJB4	DnaJ homolog subfamily B member 4	↓	-1E+10
DNAJB6	DnaJ homolog subfamily B member 6	↓	-2.02
DNAJC10	DnaJ homolog subfamily C member 10	↓	-1E+10
DNAJC11	DnaJ homolog subfamily C member 11	↑	6.74
Gstm3	Glutathione S-transferase Mu 3	↓	-7.75
KRAS	GTPase KRas	↑	2
MAP2K2	Dual specificity mitogen-activated protein kinase kinase 2	↓	-3.7
MAP2K4	Dual specificity mitogen-activated protein kinase kinase 4	↑	2.03
MAPK1	Mitogen-activated protein kinase 1	↓	-2.95
NQO2	Ribosyldihydronicotinamide dehydrogenase [quinone]	↑	2.19
RALA	Ras-related protein Ral-A	↑	2.03
RAP1A	Ras-related protein Rap-1A	↑	2.68
RAP2B	Ras-related protein Rap-2b	↓	-1E+10
RRAS2	Ras-related protein R-Ras2	↓	-1E+10
SCARB1	Scavenger receptor class B member 1	↓	-2.27
SOD3	Extracellular superoxide dismutase [Cu-Zn]	↑	1E+10
TXN	Thioredoxin	↓	-2.9

**Table 4:** The effect of the genetic deletion of *dnajb4* in the livers of *apoe^-/-^* mice on apoptosis signaling pathway-related proteins.

Protein	Protein description	*apoe^-/-^dnajb4^-/-^* vs. *apoe^-/-^*	Fold change
ACIN1	Apoptotic chromatin condensation inducer in the nucleus	↓	-13.83
CAPNS1	Calpain small subunit 1	↓	-7.64
CASP6	Caspase-6	↑	1E+10
CASP7	Caspase-7	↓	-1E+10
ENDOG	Endonuclease G, mitochondrial	↓	-2.81
GAS2	Growth arrest-specific protein 2	↓	-1E+10
KRAS	GTPase KRas	↑	2
MAP2K2	Dual specificity mitogen-activated protein kinase kinase 2	↓	-3.7
MAP2K4	Dual specificity mitogen-activated protein kinase kinase 4	↑	2.03
MAPK1	Mitogen-activated protein kinase 1	↓	-2.95
PARP1	Poly [ADP-ribose] polymerase 1	↓	-1E+10
RALA	Ras-related protein Ral-A	↑	2.03
RAP1A	Ras-related protein Rap-1A	↑	2.68
RAP2B	Ras-related protein Rap-2b	↓	-1E+10
ROCK1	Rho-associated protein kinase 1	↓	-4.02
RRAS2	Ras-related protein R-Ras2	↓	-1E+10

**Table 5:** The effect of the genetic deletion of *dnajb4* in the livers of *apoe^-/-^* mice on Rho family GTPases signaling pathway-related proteins.

Protein	Protein description	*apoe^-/-^dnajb4^-/-^* vs. *apoe^-/-^*	Fold change
ACTC1	Actin, alpha cardiac muscle 1	↑	2.62
ACTG1	Actin, cytoplasmic 2	↑	3.55
ARHGEF12	Rho guanine nucleotide exchange factor 12	↓	-2.31
ARPC5	Actin-related protein 2/3 complex subunit 5	↑	3.75
ARPC5L	Actin-related protein 2/3 complex subunit 5-like protein	↑	2.36
BAIAP2	Brain-specific angiogenesis inhibitor 1-associated protein 2	↑	14.53
CDC42	Cell division control protein 42 homolog	↑	4.69
CDH1	Cadherin-1	↓	-6.32
CFL1	Cofilin-1	↑	2.41
CLIP1	CAP-Gly domain-containing linker protein 1	↓	-2.29
EZR	Ezrin	↑	3.38
GNA13	Guanine nucleotide-binding protein subunit alpha-13	↑	1E+10
IQGAP1	Ras GTPase-activating-like protein IQGAP1	↓	-2.82
ITGA9	Integrin alpha-9	↓	-2.81
ITGAV	Integrin alpha-V	↓	-31.66
ITGB3	Integrin beta-3	↓	-4.49
MAP2K2	Dual specificity mitogen-activated protein kinase kinase 2	↓	-3.7
MAP2K4	Dual specificity mitogen-activated protein kinase kinase 4	↑	2.03
MAPK1	Mitogen-activated protein kinase 1	↓	-2.95
MSN	Moesin	↓	-2.84
MYL6	Myosin light polypeptide 6	↑	1E+10
PAK2	Serine/threonine-protein kinase PAK 2	↓	-1E+10
PI4KA	Phosphatidylinositol 4-kinase alpha	↑	1E+10
RHOT2	Mitochondrial Rho GTPase 2	↓	-3.04
ROCK1	Rho-associated protein kinase 1	↓	-4.02
SEPTIN11	Septin-11	↓	-1E+10

**Table 6:** The effect of the genetic deletion of *dnajb4* in the livers of *apoe^-/-^* mice on mitochondrial dysfunction pathway-related proteins.

Protein	Protein description	*apoe^-/-^dnajb4^-/-^* vs. *apoe^-/-^*	Fold change
ATP5MG	ATP synthase subunit g, mitochondrial	↑	2.55
CYB5B	Cytochrome b5 type B	↑	2.12
DHODH	Dihydroorotate dehydrogenase (quinone), mitochondrial	↓	-1E+10
FIS1	Mitochondrial fission 1 protein	↑	2.44
HSD17B10	3-hydroxyacyl-CoA dehydrogenase type-2	↑	2.4
MAP2K4	Dual specificity mitogen-activated protein kinase kinase 4	↑	2.03
NDUFA4	Cytochrome c oxidase subunit NDUFA4	↑	2.7
NDUFA9	NADH dehydrogenase [ubiquinone] 1 alpha subcomplex subunit 9, mitochondrial	↑	2.01
NDUFB10	NADH dehydrogenase [ubiquinone] 1 beta subcomplex subunit 10	↑	2.53
NDUFB4	NADH dehydrogenase [ubiquinone] 1 beta subcomplex subunit 4	↑	4.91
PRDX5	Peroxiredoxin-5, mitochondrial	↑	2.36
RHOT2	Mitochondrial Rho GTPase 2	↓	-3.04
SDHC	Succinate dehydrogenase cytochrome b560 subunit, mitochondrial	↑	2.02
SURF1	Surfeit locus protein 1	↑	1E+10
TXNRD2	Thioredoxin reductase 2, mitochondrial	↓	-3.28
UQCR10	Cytochrome b-c1 complex subunit 9	↑	2.98
UQCRB	Cytochrome b-c1 complex subunit 7	↑	2.04
UQCRFS1	Cytochrome b-c1 complex subunit Rieske, mitochondrial	↑	2.41
UQCRQ	Cytochrome b-c1 complex subunit 8	↑	1E+10
VDAC3	Voltage-dependent anion-selective channel protein 3	↓	-3.5

**Table 7:** The effect of the genetic deletion of *dnajb4* in the livers of *apoe^-/-^* mice on LXR/RXR activation pathway-related proteins.

Protein	Protein description	*apoe^-/-^dnajb4^-/-^* vs. *apoe^-/-^*	Fold change
AGT	Angiotensinogen	↑	1E+10
AHSG	Alpha-2-HS-glycoprotein	↓	-2.44
AMBP	Protein AMBP	↓	-1E+10
APOA4	Apolipoprotein A-IV	↓	-1E+10
APOB	Apolipoprotein B-100	↑	2.05
APOE	Apolipoprotein E	↑	1E+10
APOH	Beta-2-glycoprotein 1	↓	-2.6
CD36	Platelet glycoprotein 4	↓	-3.57
CLU	Clusterin	↓	-2.65
FDFT1	Squalene synthase	↓	-3.91
ITIH4	Inter alpha-trypsin inhibitor, heavy chain 4	↓	-6.11
KNG1	Kininogen-1	↓	-3.04
LYZ	Lysozyme C-1	↓	-6.8
RBP4	Retinol-binding protein 4	↑	3.34
SERPINF2	Alpha-2-antiplasmin	↓	-2.27
TF	Serotransferrin	↓	-2.68

**Table 8:** The effect of the genetic deletion of *dnajb4* in the livers of *apoe^-/-^* mice on tight junction signaling pathway-related proteins.

Protein	Protein description	*apoe^-/-^dnajb4^-/^*^-^ vs. *apoe*^-/-^	Fold change
ACTC1	Actin, alpha cardiac muscle 1	↑	2.62
ACTG1	Actin, cytoplasmic 2	↑	3.55
AFDN	Afadin	↓	-6.65
EPB41	Protein 4.1	↓	-26.88
MPDZ	Multiple PDZ domain protein	↓	-1E+10
MYH1	Myosin-1	↓	-1E+10
MYH11	Myosin-11	↑	1E+10
MYL6	Myosin light polypeptide 6	↑	1E+10
NAPG	Gamma-soluble NSF attachment protein	↓	-1E+10
NUDT21	Cleavage and polyadenylation specificity factor subunit 5	↑	8.13
PPP2R2A	Serine/threonine-protein phosphatase 2A 55 kDa regulatory subunit B alpha isoform	↑	3.39
PPP2R5A	Serine/threonine-protein phosphatase 2A 56 kDa regulatory subunit alpha isoform	↑	2.65
PRKACB	cAMP-dependent protein kinase catalytic subunit beta	↓	-30.65
PRKAR2A	cAMP-dependent protein kinase type II-alpha regulatory subunit	↓	-14.57
TJP2	Tight junction protein ZO-2	↓	-9.29
TJP3	Tight junction protein ZO-3	↓	-1E+10
VAPA	Vesicle-associated membrane protein-associated protein A	↓	-3.89
YKT6	Synaptobrevin homolog YKT6	↑	3.79

**Table 9:** The effect of the genetic deletion of *dnajb4* in the livers of *apoe*^-/-^ mice on the superpathway of cholesterol biosynthesis-related proteins.

Protein	Protein description	*apoe^-/-^dnajb4^-/-^* vs.* apoe^-/-^*	Fold change
FDFT1	Squalene synthase	↓	-3.91
HMGCS1	Hydroxymethylglutaryl-CoA synthase, cytoplasmic	↓	-4.48
IDI1	Isopentenyl-diphosphate Delta-isomerase 1	↓	-2.79
LSS	Lanosterol synthase	↓	-4.21
MVD	Diphosphomevalonate decarboxylase	↓	-1E+10
PMVK	Phosphomevalonate kinase	↓	-1E+10
TM7SF2	Delta(14)-sterol reductase TM7SF2	↑	1E+10

**Table 10:** The effect of the genetic deletion of *dnajb4* in the livers of *apoe*^-/-^ mice on oxidative phosphorylation-related proteins.

Protein	Protein description	*apoe^-/-^dnajb4^-/-^* vs*. apoe^-/-^*	Fold change
ATP5MG	ATP synthase subunit g, mitochondrial	↑	2.55
CYB5B	Cytochrome b5 type B	↑	2.12
NDUFA4	Cytochrome c oxidase subunit NDUFA4	↑	2.7
NDUFA9	NADH dehydrogenase [ubiquinone] 1 alpha subcomplex subunit 9, mitochondrial	↑	2.01
NDUFB10	NADH dehydrogenase [ubiquinone] 1 beta subcomplex subunit 10	↑	2.53
NDUFB4	NADH dehydrogenase [ubiquinone] 1 beta subcomplex subunit 4	↑	4.91
SDHC	Succinate dehydrogenase cytochrome b560 subunit, mitochondrial	↑	2.02
SURF1	Surfeit locus protein 1	↑	1E+10
UQCR10	Cytochrome b-c1 complex subunit 9	↑	2.98
UQCRB	Cytochrome b-c1 complex subunit 7	↑	2.04
UQCRFS1	Cytochrome b-c1 complex subunit Rieske, mitochondrial	↑	2.41
UQCRQ	Cytochrome b-c1 complex subunit 8	↑	1E+10

## References

[1] Kim H.Y (2022). and S. Hong. Multi-Faceted Roles of DNAJB Protein in Cancer Metastasis and Clinical Implications. Int J Mol Sci.

[2] Kampinga H.H (2010). and E.A. Craig. The HSP70 chaperone machinery: J proteins as drivers of functional specificity. Nat Rev Mol Cell Biol.

[3] Rosenzweig R (2019). The Hsp70 chaperone network. Nat Rev Mol Cell Biol.

[4] Johnson O.T (2022). Two distinct classes of cochaperones compete for the EEVD motif in heat shock protein 70 to tune its chaperone activities. J Biol Chem.

[5] Tsai M.F (2006). A new tumor suppressor DnaJ-like heat shock protein, HLJ1, and survival of patients with non-small-cell lung carcinoma. J Natl Cancer Inst.

[6] Chen Y (2023). DNAJB4 suppresses breast cancer progression and promotes tumor immunity by regulating the Hippo signaling pathway. Discov Oncol.

[7] Wang C.C (2005). The transcriptional factor YY1 upregulates the novel invasion suppressor HLJ1 expression and inhibits cancer cell invasion. Oncogene.

[8] Liu Y (2014). HLJ1 is a novel biomarker for colorectal carcinoma progression and overall patient survival. Int J Clin Exp Pathol.

[9] Badimon L (2012). and G. Vilahur. LDL-cholesterol versus HDL-cholesterol in the atherosclerotic plaque: inflammatory resolution versus thrombotic chaos. Ann N Y Acad Sci.

[10] Libby P (2001). Managing the risk of atherosclerosis: the role of high-density lipoprotein. Am J Cardiol.

[11] Badmus O.O (2022). Molecular mechanisms of metabolic associated fatty liver disease (MAFLD): functional analysis of lipid metabolism pathways. Clin Sci (Lond).

[12] Nemes K (2016). Cholesterol metabolism in cholestatic liver disease and liver transplantation: From molecular mechanisms to clinical implications. World J Hepatol.

[13] Tangirala R.K (2002). Identification of macrophage liver X receptors as inhibitors of atherosclerosis. Proc Natl Acad Sci U S A.

[14] Teupser D (2008). Effect of macrophage overexpression of murine liver X receptor-alpha (LXR-alpha) on atherosclerosis in LDL-receptor deficient mice. Arterioscler Thromb Vasc Biol.

[15] Savla S.R, K.S (2022). Prabhavalkar, and L.K. Bhatt. Liver X receptor: a potential target in the treatment of atherosclerosis. Expert Opin Ther Targets.

[16] Wang B (2018). and P. Tontonoz. Liver X receptors in lipid signalling and membrane homeostasis. Nat Rev Endocrinol.

[17] Laffitte B.A (2001). Autoregulation of the human liver X receptor alpha promoter. Mol Cell Biol.

[18] Gungor B (2019). HSP70 induces liver X receptor pathway activation and cholesterol reduction *in vitro* and *in vivo*. Mol Metab.

[19] Chen H (2008). W, et al. Curcumin inhibits lung cancer cell invasion and metastasis through the tumor suppressor HLJ1. Cancer research.

[20] Lai Y (2013). H, et al. The HLJ1-targeting drug screening identified Chinese herb andrographolide that can suppress tumour growth and invasion in non-small-cell lung cancer. Carcinogenesis.

[21] Szklarczyk D (2023). The STRING database in 2023: protein-protein association networks and functional enrichment analyses for any sequenced genome of interest. Nucleic acids research.

[22] Li H (2021). Hepatic cholesterol transport and its role in non-alcoholic fatty liver disease and atherosclerosis. Prog Lipid Res.

[23] Ciriaci N, P.-E (2024). Rautou, and J. Poisson. Loss of fenestrae in liver sinusoidal endothelial cells contributes to MASLD. Nature Cardiovascular Research.

[24] Park S.J (2023). Major roles of kupffer cells and macrophages in NAFLD development. Front Endocrinol (Lausanne).

[25] Xu G.X (2023). Activation of Kupffer cells in NAFLD and NASH: mechanisms and therapeutic interventions. Front Cell Dev Biol.

[26] Chen C.H (2022). Ractopamine at legal residue dosage accelerates atherosclerosis by inducing endothelial dysfunction and promoting macrophage foam cell formation. Environ Pollut.

[27] Lee T.S (2021). Hyperuricemia induces endothelial dysfunction and accelerates atherosclerosis by disturbing the asymmetric dimethylarginine/dimethylarginine dimethylaminotransferase 2 pathway. Redox Biol.

[28] Panjwani N.N (2002). Heat shock proteins gp96 and hsp70 activate the release of nitric oxide by APCs. J Immunol.

[29] Jacquier-Sarlin M.R (1994). Protective effects of hsp70 in inflammation. Experientia.

[30] Lazarev V.F (2016). Hsp70 chaperone rescues C6 rat glioblastoma cells from oxidative stress by sequestration of aggregating GAPDH. Biochem Biophys Res Commun.

[31] Rampelt H (2012). Metazoan Hsp70 machines use Hsp110 to power protein disaggregation. Embo j.

[32] Liu Y.N (2008). YC-1 induces heat shock protein 70 expression and prevents oxidized LDL-mediated apoptosis in vascular smooth muscle cells. Shock.

[33] Bielecka-Dabrowa A (2009). HSP 70 and atherosclerosis-protector or activator?. Expert Opin Ther Targets.

[34] Peet DJ (1998). Cholesterol and bile acid metabolism are impaired in mice lacking the nuclear oxysterol receptor LXR alpha. Cell.

[35] Laffitte B.A (2003). Activation of liver X receptor improves glucose tolerance through coordinate regulation of glucose metabolism in liver and adipose tissue. Proc Natl Acad Sci U S A.

[36] Zhou E (2023). Inhibition of DHCR24 activates LXRα to ameliorate hepatic steatosis and inflammation. EMBO Mol Med.

[37] Mayer M.P (2019). and L.M. Gierasch. Recent advances in the structural and mechanistic aspects of Hsp70 molecular chaperones. J Biol Chem.

[38] Balchin D (2016). *In vivo* aspects of protein folding and quality control. Science.

[39] Hager GL (2004). Dynamics of nuclear receptor movement and transcription. Biochim Biophys Acta.

[40] Pratt WB, Toft DO (1997). Steroid receptor interactions with heat shock protein and immunophilin chaperones. Endocr Rev.

[41] Faust O (2020). HSP40 proteins use class-specific regulation to drive HSP70 functional diversity. Nature.

[42] Frydman J (2001). Folding of newly translated proteins *in vivo*: the role of molecular chaperones. Annu Rev Biochem.

[43] Żwirowski S (2017). Hsp70 displaces small heat shock proteins from aggregates to initiate protein refolding. EMBO J.

[44] Zietkiewicz S (2006). Hsp70 chaperone machine remodels protein aggregates at the initial step of Hsp70-Hsp100-dependent disaggregation. J Biol Chem.

[45] Li L (2023). Nuclear import carrier Hikeshi cooperates with HSP70 to promote murine oligodendrocyte differentiation and CNS myelination. Dev Cell.

[46] Prasad R, C (2018). Xu, and D.T.W. Ng. Hsp40/70/110 chaperones adapt nuclear protein quality control to serve cytosolic clients. J Cell Biol.

