# Supplementary material for: DNAJB4 Protects Against Atherosclerosis by Stabilizing the HSP70-LXRα Axis in Hepatic Lipid Metabolism
Source: Int J Biol Sci. 2026 Aug 21;22(13):7380–400. doi: 10.7150/ijbs.131807 (PMC13541000; doi:10.7150/ijbs.131807)
Supplement: Supplementary file 1 [file ijbsv22p7380s1.pdf]

Supplementary Materials

DNAJB4 Protects Against Atherosclerosis by Stabilizing the HSP70-LXRα Axis in Hepatic Lipid Metabolism

Wen-Hua Chen[1,#], Chia-Hui Chen[1,#], Man-Chen Hsu[1], Po-An Hu[1], Hsueh-Te Lee[2,3], Hsuan-Yun Hu[2,3], Kang-Yi Su[4,5], Wei-Lun Hsu[4], Tzong-Shyuan Lee[1,*]

#, these authors equally contribute to this work

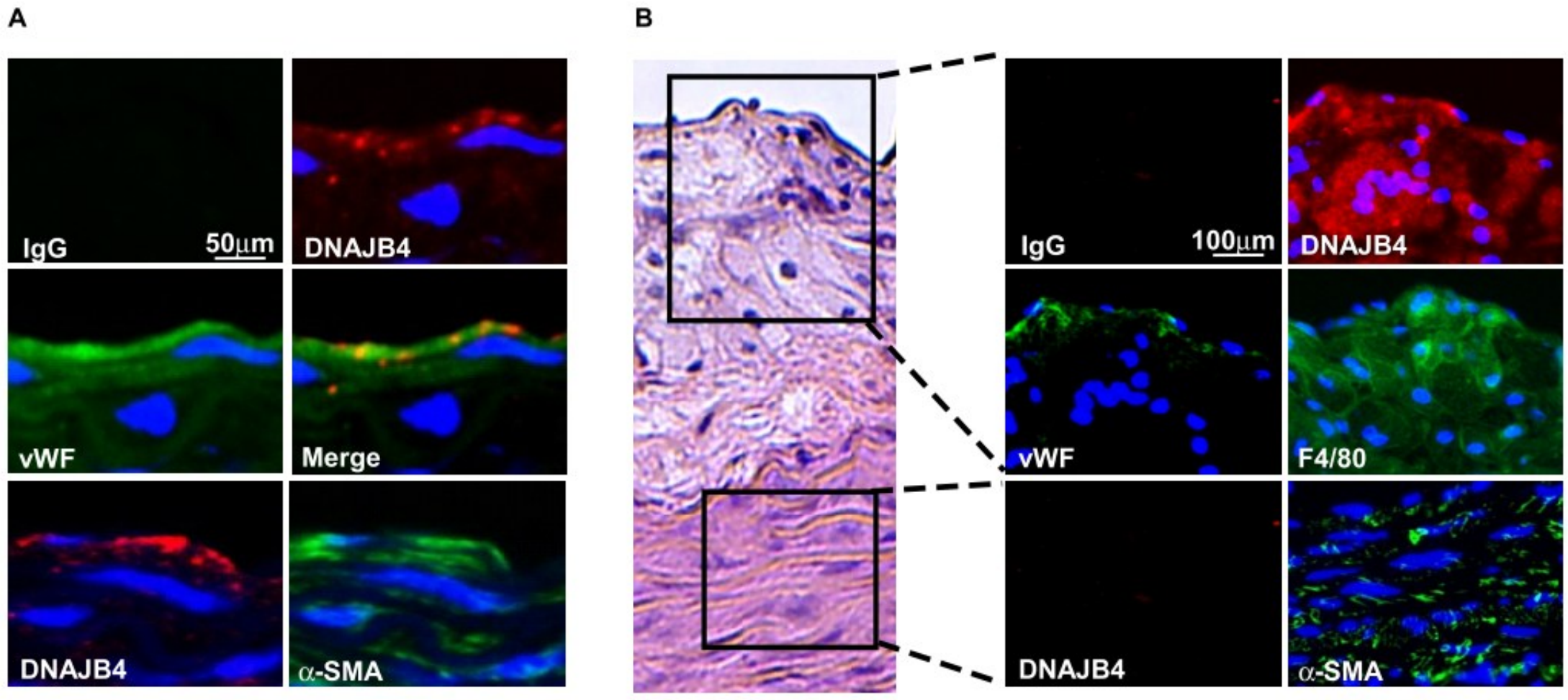

Figure S1. Cellular distribution of DNAJB4 in normal aortas and atherosclerotic aortas. Aortas were collected from 5-month-old (A) WT mice or (B) apoE$^{-/-}$ mice and subjected to immunohistochemistry. Immunostaining with normal rabbit IgG, anti-DNAJB4, anti-vWF (an EC marker), anti-F4/80 (a macrophage marker) or anti-α-smooth muscle actin (a SMC marker) was performed. Cellular nuclei were stained with DAPI.

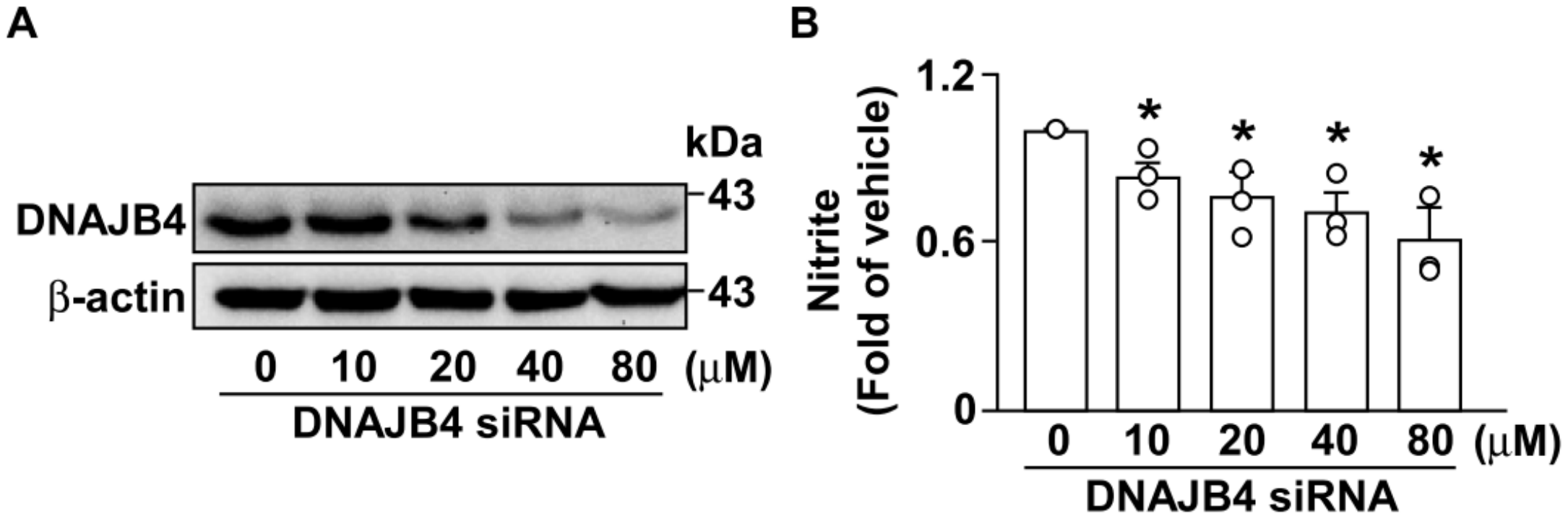

Figure S2. The absence of DNAJB4 decreases nitrite production in human microvascular endothelial cells (HMECs). HMECs were transfected with DNAJB4 siRNA (100 nM) for 18 hr. (A) Western blot analysis of DNAJB4. (B) Nitrite production was examined by Griess reagent. Data are expressed as the mean ± SEM from three independent experiments (n=3). *$p < 0.05$ vs. vehicle group.

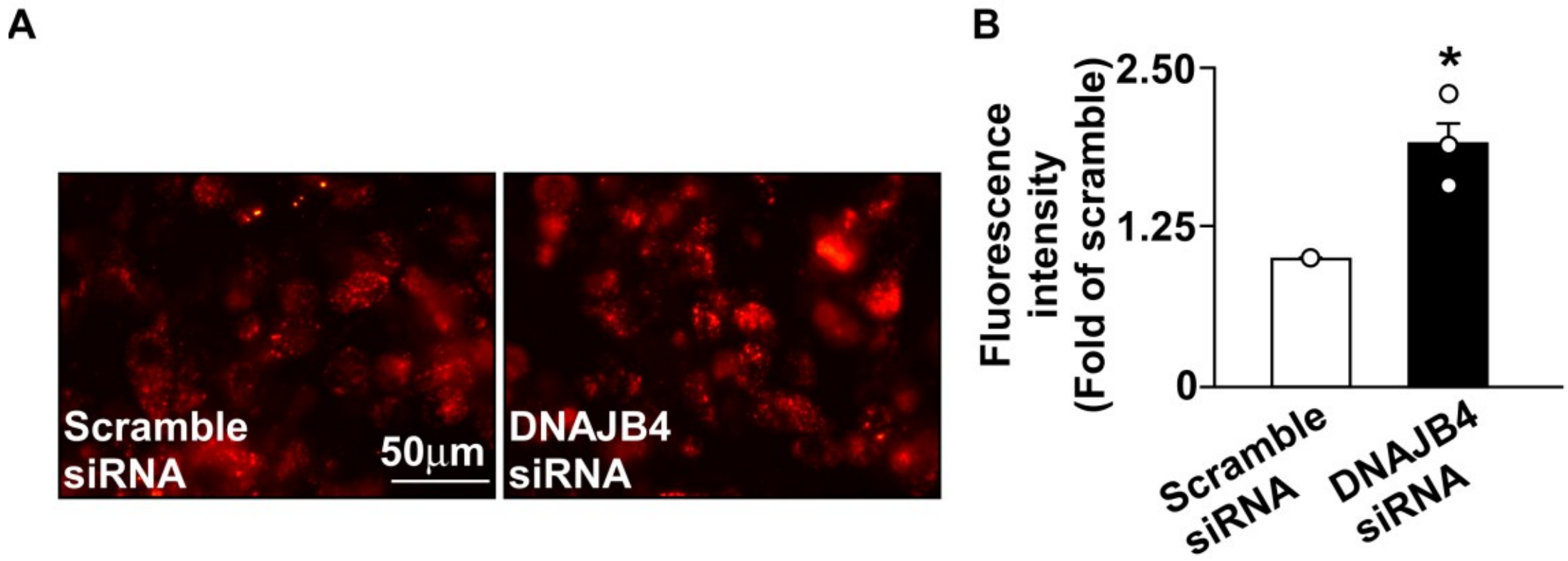

Figure S3. The absence of DNAJB4 exacerbates the oxidized LDL accumulation in macrophages. THP-1 cells were induced with 50 nM PMA for five days to differentiate into macrophages, and then transfected with DNAJB4 siRNA (100 nM). Macrophages were subsequently treated with dil-oxidized LDL (50 mg/ml) for 18 h. (A and B) Representative fluorescent images and fluorescence intensity of lipid accumulation. Data are expressed as the mean ± SEM from three independent experiments (n=3). *$p < 0.05$ vs. vehicle group.
